# A Comprehensive Review of Na_4_Fe_3_(PO_4_)_2_P_2_O_7_ Cathode Materials for Sodium-Ion Batteries: From Crystal Structure and Phase Purification to Modification Strategies Progress

**DOI:** 10.3390/molecules31183153

**Published:** 2026-09-08

**Authors:** Yong-Gang Sun, Jian Xiong, Xiang-Yu Qian, Jin-Yi Ding, Yi-Han Zhang, Li Dong, Yu Hu, Xin Wang, Bei-Bei Zhang, Feng-Cai Li, Song Chen

**Affiliations:** School of Chemistry and Chemical Engineering, Yancheng Institute of Technology, Yancheng 224051, China; sunyg86@iccas.ac.cn (Y.-G.S.); qianxy05@outlook.com (X.-Y.Q.); djy11062026@163.com (J.-Y.D.); ruarua0505@163.com (Y.-H.Z.); dongli@stu.ycit.edu.cn (L.D.); huyu@stu.ycit.edu.cn (Y.H.); wang92643864@163.com (X.W.); yizhou1118@163.com (B.-B.Z.); jsyccs@163.com (S.C.)

**Keywords:** sodium-ion battery, Na_4_Fe_3_(PO_4_)_2_P_2_O_7_ cathode, Phase Adjustment, elemental doping, defect engineering, heterostructure design

## Abstract

Na_4_Fe_3_(PO_4_)_2_P_2_O_7_ (NFPP), an iron-based mixed phosphate–pyrophosphate cathode material, has emerged as one of the most commercially promising candidates for large-scale sodium-ion battery (SIB) energy storage applications. Its exceptional characteristics—an ultralow volume change of less than 4% during Na^+^ de/intercalation, a three-dimensional open framework enabling rapid ionic diffusion, and the use of earth-abundant, low-cost iron as the redox center—collectively deliver a unique combination of structural stability, rate capability, and economic viability. However, the fundamental challenge of phase-purity control, arising from the three-phase thermodynamic competition among NFPP, electrochemically inert maricite-NaFePO_4_, and Na_2_FeP_2_O_7_ during synthesis, critically limits its electrochemical performance. This review provides a systematic overview of NFPP research progress from 2012 to 2026, covering crystal structure and sodium storage mechanisms, synthesis methodologies, and—most critically—Phase Adjustment and modification strategies including non-stoichiometric regulation, defect engineering, elemental doping, anionic substitution, and heterostructure design. Mechanistic insights into how each strategy addresses the phase-purity challenge and enhances electrochemical kinetics are critically examined. Industrialization progress, full-cell performance evaluation, cost analysis, and future research directions toward practical deployment are also discussed.

## 1. Introduction

The global energy transition and the drive toward carbon neutrality have made large-scale electrochemical energy storage indispensable [1,2,3]. It underpins high-penetration renewable energy integration and the safe operation of smart grids. Lithium-ion batteries (LIBs) currently dominate the market [4,5,6]. However, concerns about their long-term sustainability are mounting. Roughly 70% of global lithium reserves are concentrated in the South American “lithium triangle,” creating supply risks and persistent cost volatility [7,8,9,10,11]. Sodium-ion batteries (SIBs) present a compelling alternative [12,13,14,15,16]. Sodium is vastly more abundant in the Earth crust—2.36% versus 0.0017% for lithium—and is distributed globally [17,18,19,20]. SIBs also operate on the same rocking-chair principle as LIBs. These advantages position SIBs as one of the most competitive replacement technologies for grid-scale energy storage and low-speed electric vehicles [21,22,23,24,25,26].

Among the many SIB cathode families, polyanionic compounds have attracted intense interest [27,28,29,30]. Their strong covalent frameworks deliver outstanding structural stability, thermal safety, and long cycle life [31,32,33,34,35,36,37]. Within this family, the iron-based mixed phosphate–pyrophosphate Na_4_Fe_3_(PO_4_)_2_P_2_O_7_ (NFPP) has distinguished itself since Kim et al. [38] first reported its electrochemical sodium storage performance in 2012. Several unique advantages set NFPP apart. (i) A three-dimensional open framework built from [Fe_3_(PO_4_)_2_] layers and P_2_O_7_ dimers creates interconnected fast Na^+^ diffusion channels [39,40]. (ii) An ultralow lattice volume change below 4% during Na^+^ (de)intercalation imparts a near-zero-strain character, providing the structural foundation for exceptionally long cycle life [41]. (iii) Iron—the most abundant and cheapest transition metal in the Earth crust—serves as the sole redox center, yielding a decisive raw-material cost advantage [42,43]. (iv) The strong inductive effect of the mixed PO4^3−^/P_2_O_7_^4−^ polyanionic framework elevates the Fe^2+^/Fe^3+^ redox potential to roughly 3.0 V (vs. Na^+^/Na), significantly outperforming single-phosphate systems [44].

Yet NFPP faces a fundamental bottleneck on the path to practical application. During synthesis, three phases compete thermodynamically—the target NFPP, electrochemically inert maricite-type NaFePO_4_, and Na_2_FeP_2_O_7_—making precise phase-purity control extremely difficult [45]. Maricite adopts a dense, close-packed crystal structure. It completely lacks the open Na^+^ diffusion channels that NFPP possesses [46]. Its presence degrades performance through two mechanisms. First, it dilutes the gravimetric specific capacity. More critically, it blocks ion transport pathways and disrupts the electronic percolation network, causing a disproportionate collapse in rate capability [47]. Breaking this thermodynamic bottleneck to reach the controlled synthesis of high-phase-purity NFPP is thus become the central scientific question that must be resolved for practical deployment [48,49,50,51].

**Figure 1:**
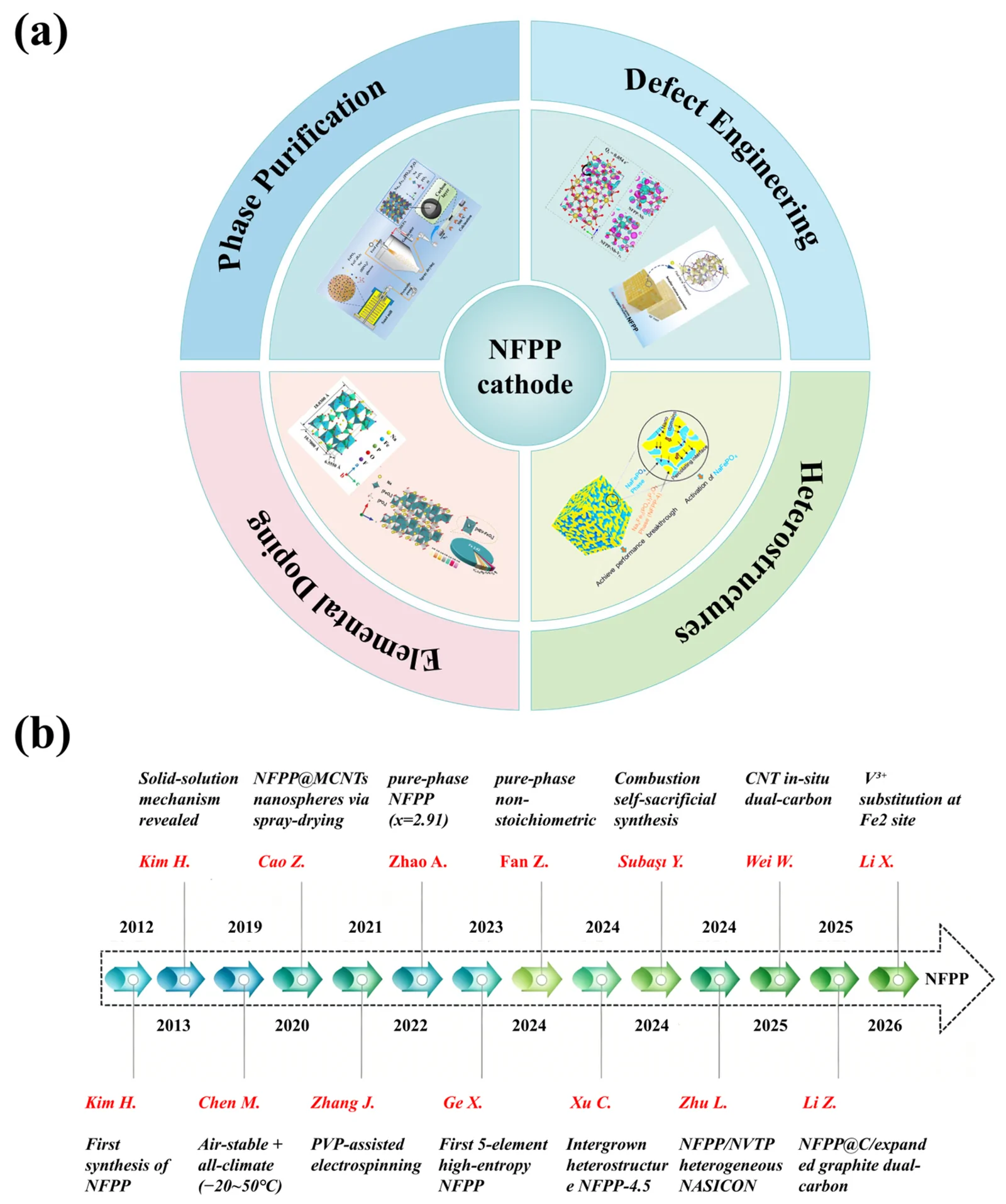
(**a**) Schematic NFPP modification strategies; (**b**) Historical evolution of NFPP research.

Addressing this central challenge of phase-purity control, researchers have developed an increasingly rich portfolio of modification strategies. These span non-stoichiometric regulation [52], defect engineering [53,54] (Fe/Na/O vacancies), cation doping [55,56,57,58,59,60] (high-valence V/Al/Ti/Zr/Mo/Nb, low-valence Mn/Ni, and high-entropy multi-element doping), anion substitution [61,62,63] (SiO_4_^4−^), F^−^, and F/Si co-doping), and heterostructure/dual-phase design [64,65]. Since 2023 in particular, a series of breakthroughs have propelled the comprehensive performance of NFPP to unprecedented levels. These include the entropy stabilization effect of high-entropy doping, donor-level engineering of oxygen vacancies, precise tuning of the V^3+^ coordination environment, and interfacial synergy in intergrowth heterostructures. The record reversible capacity has surged from roughly 120 mAh g^−1^ [55] to 150.7 mAh g^−1^ [66]. Cycle life has exceeded 15,000 cycles [67]. Ah-level pouch cells have achieved stable cycling validation [68]. The panoramic landscape of NFPP modification strategies and the historical evolution of the research field is summarized in Figure 1.

This review systematically surveys the fundamental research and technological progress of NFPP cathode materials from 2012 to 2026. In Section 2, the crystal structure, Na^+^ (de)intercalation mechanism, and principal synthesis methods are described. The working mechanisms, key breakthroughs, and structure–performance relations of each modification strategy are considered in Section 3. It follows a progressive logic from Phase Adjustment to defect engineering, elemental doping, anion substitution, and finally heterostructure design. The current research status and outlines future directions across five dimensions: rational Phase Adjustment, multi-scale design, extreme-condition performance, sustainability, and cross-system extension are summarized in Section 4. While several recent reviews have surveyed specific aspects of NFPP [69,70]—crystal chemistry, synthesis, and scale-up/industrialization—the present review integrates all major modification strategies under the unifying thread of phase-purity control and traces their evolution from empirical trial-and-error to mechanism-guided design.

## 2. Crystal Structure, Sodium Storage Mechanism, and Synthesis Methods

### 2.1. Crystal Structure Characteristics

**Figure 2:**
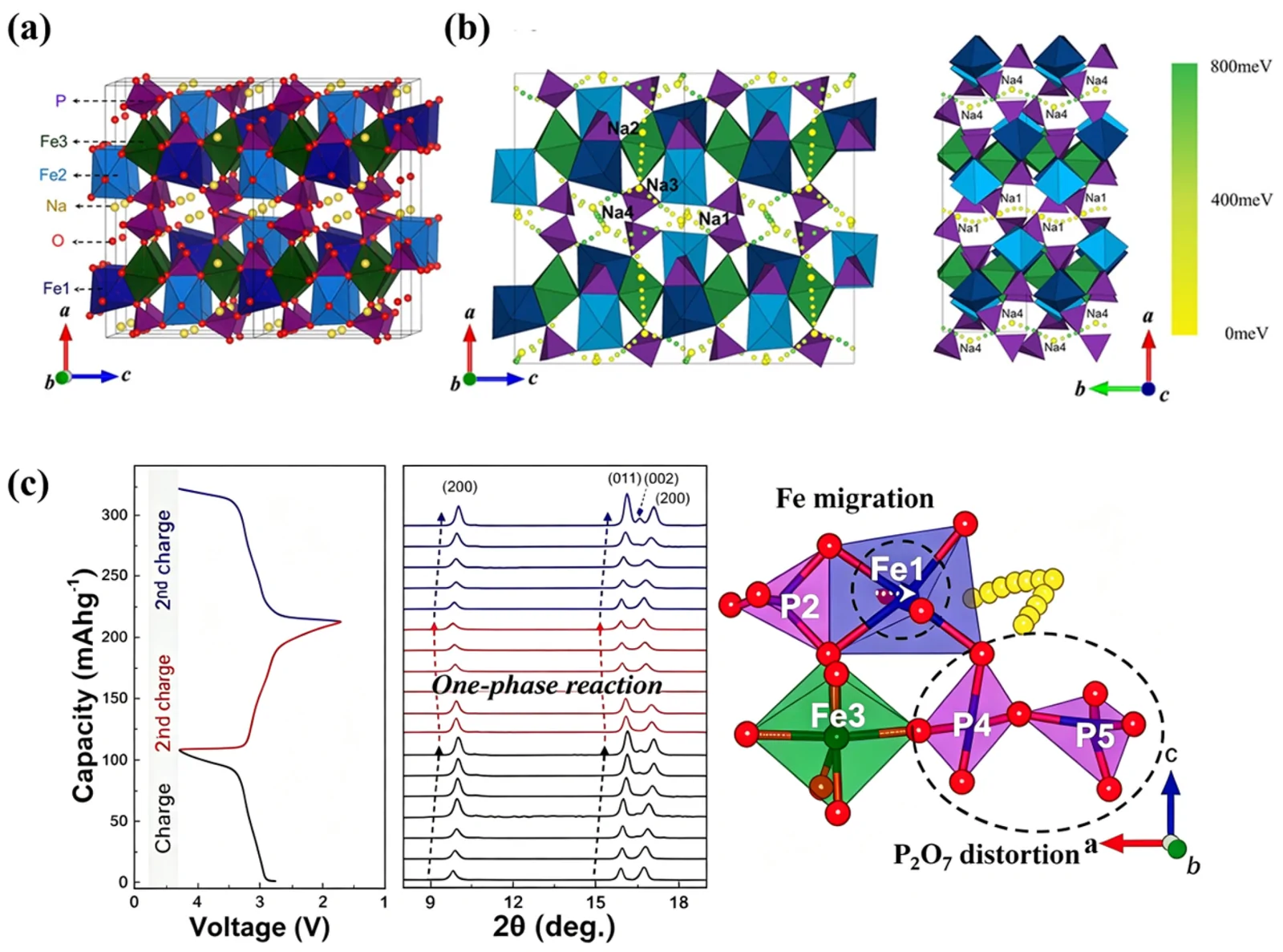
(**a**) a–c plane projection of NFPP along the b−axis; (**b**) schematic of the different Na site structures [38]; (**c**) Na^+^ storage mechanism in NFPP [72].

NFPP crystallizes in an orthorhombic system, space group *Pn*2_1_*a* (No. 33). The unit-cell parameters are a ≈ 18.05 Å, b ≈ 6.57 Å, c ≈ 10.69 Å, V ≈ 1268 Å^3^. The structure was first reported by Sanz et al. [71] and subsequently refined by Kim et al. [38]. The NFPP framework can be considered as a combination of two fundamental building units: [Fe_3_P_2_O_13_] slabs parallel to the a–c plane and P2O7 pyrophosphate dimers bridging adjacent layers along the b-axis [72,73,74] (Figure 2a). Together they construct an open three-dimensional network. Within the [Fe_3_P_2_O_13_] slabs, three crystallographically independent FeO_6_ octahedra (Fe1, Fe2, and Fe3 sites) corner-share with PO_4_ tetrahedra, producing a dense polyhedral connectivity that imparts structural rigidity to the framework. P_2_O_7_ dimers consist of two corner-sharing PO_4_ tetrahedra whose bent P–O–P linkage grants the pyrophosphate group conformational flexibility. Studies indicate that P_2_O_7_ bending vibrations and translational modes may dynamically assist Na^+^ migration along the channels, producing a “paddlewheel” effect [75,76].

A key structural feature of NFPP is the presence of four crystallographically independent Na^+^ sites (Na1–Na4) distributed throughout the 3D framework [38] (Figure 2b). Their coordination environments range from six-coordinate octahedral to eight-coordinate polyhedral geometries, yielding distinct Na^+^ binding energies and extraction potentials. The Na^+^ sites are interconnected by a three-dimensional diffusion network. This network comprises one-dimensional main channels along the b-axis and two-dimensional cross-channels lying in the a–c plane. This 3D interconnectivity is critical for rate performance, providing multiple alternative diffusion pathways that mitigate channel blockage caused by defects or impurity phases [77,78,79,80]. Meanwhile, the strong P–O covalent bonds in PO_4_^3−^ and P2O7^4−^ groups exert a pronounced inductive effect that withdraws electron density from Fe–O bonds [72] (Figure 2c). This raises the Fe^2+^/Fe^3+^ redox potential to approximately 3.0 V (vs. Na^+^/Na), about 0.4 V above that of olivine NaFePO_4_. Electronic band structure calculations revealed a band gap of approximately 2.99 eV, confirming insulating character of NFPP and the necessity of conductive carbon coating for electrochemical function. The crystal structure, Na^+^ site distribution, and sodium storage mechanism are shown in Figure 2.

### 2.2. Na^+^ (De)intercalation Mechanism

The electrochemical sodiation/desodiation of NFPP proceeds via a non-ideal solid-solution reaction mechanism. This stands in stark contrast to the typical two-phase reactions observed in olivine NaFePO_4_ and many NASICON-type cathodes [81,82,83,84]. Kim et al. and Wu et al. [84] identified, via in situ XRD, two main voltage plateaus at ~2.9 V and ~3.2 V (vs. Na^+^/Na). These plateaus reflect the sequential extraction of Na^+^ from four crystallographic sites with distinct binding energies. During desodiation, Na^+^ is first extracted from the Na4 site, which exhibits the weakest binding and accounts for the ~2.9 V plateau. Extraction then proceeds from Na1 and Na2 sites, corresponding to the ~3.2 V plateau. Meanwhile, Na^+^ at the Na3 site remains partially occupied throughout cycling, serving as a structural “pillar” that prevents framework collapse. This sequential extraction, combined with the solid-solution-type phase transition, avoids sharp volume discontinuities characteristic of first-order transformations. It is a key contributor to the exceptional cycling stability of NFPP.

Anisotropic lattice parameter evolution during charge/discharge is a defining characteristic of NFPP. In situ/operando XRD measurements reveal that the a-axis contracts by ~4% while the c-axis elongates slightly, yielding a total unit-cell volume change below 4%. This approaches “zero-strain” cathode territory, far smaller than the 6–10% volume changes typical of layered oxide cathodes [38,85]. Such minimal volume change suppresses mechanical strain at both particle and electrode levels, inhibiting particle cracking and electrical contact loss. This is the primary structural basis for NFPP’s ultra-long cycling stability exceeding 10,000 cycles. BVEL calculations and DFT-based NEB methods have identified preferential Na^+^ migration pathways and quantified the associated diffusion barriers [86]. In pristine NFPP, the lowest Na^+^ hopping barrier between adjacent sites is approximately 0.50–0.53 eV [55]. The rate-limiting step involves passage through bottleneck regions defined by the oxygen coordination environment between adjacent Na sites [87,88,89]. Measurements by GITT and EIS yield D(Na^+^) values for carbon-coated NFPP typically in the range of 10^−12^ to 10^−10^ cm^2^ s^−1^. These values are competitive with NVP and NMMP cathodes [76,90,91,92,93,94,95].

### 2.3. Comparison of Major Synthesis Methods

Synthesizing phase-pure, uniformly carbon-coated NFPP requires precise control over precursor chemistry, heat-treatment parameters, and atmospheric conditions. Six mainstream synthetic routes were developed, each with distinct advantages and limitations.

Solid-state synthesis is the most viable route for industrial-scale production. In a typical procedure, stoichiometric sodium sources (Na_2_CO_3_, NaH_2_PO_4_), iron sources (FeC_2_O_4_·2H_2_O, FePO_4_), and carbon precursors (glucose, sucrose, citric acid) are mixed by high-energy ball milling. The mixture is then calcined at 550–700 °C under inert (Ar) or mildly reducing (Ar/H_2_) atmosphere [96]. This method offers low cost, simple processing, and direct scalability to ton-level production. However, it also faces challenges in achieving atomic-scale homogeneity of precursors and uniform carbon coating on the final product. Solid-state products typically exhibit particle sizes of 500 nm to 5 μm. Electrochemical performance is highly sensitive to ball-milling duration, calcination temperature ramp rate, and the type of carbon source [97,98].

The sol–gel method employs chelating agents (typically citric acid or ethylene glycol) to form a homogeneous precursor gel. Na^+^, Fe^2+^, PO4^3−^, and P_2_O_7_^4−^ ions are intimately mixed at the molecular scale, enabling lower calcination temperatures (500–600 °C) and finer, more uniform particle sizes (200–500 nm) [84]. In situ carbonization of the chelating agent during calcination produces a conformal carbon coating layer with superior uniformity compared to solid-state methods [99]. However, this method suffers from high solvent consumption, low per-batch yield (typically gram-scale), and high waste-liquid treatment costs, which pose challenges for industrial scale-up.

Spray drying strikes an optimal balance between scalability (kilogram-scale per batch) and particle morphology control. Atomized precursor droplets are dried in a hot gas stream to form spherical secondary particles (5–16 μm) assembled from nanoscale primary crystallites. This hierarchical architecture simultaneously optimizes tap density and electrolyte wettability [54,100]. The spray-drying route has emerged as the leading candidate for commercial NFPP production, with several Chinese manufacturers already adopting it for pilot-scale manufacturing. Huang et al. synthesized NFPP@CNT hollow microspheres via spray drying and reached a reversible capacity of 103.9 mAh g^−1^ at 0.1C with 99.9% capacity retention after 1000 cycles at 5C, convincingly demonstrating the method’s advantages [28].

Several alternative synthesis routes complement spray drying, each carrying distinct trade-offs. Solution combustion exploits the exothermic redox reaction between organic fuels (e.g., urea, glycine) and nitrate oxidizers to drive rapid self-sustaining crystallization, yielding ultrafine nanoparticles (50–200 nm). Precise control of the fuel-to-oxidizer ratio is essential to suppress excessive grain growth [101]. Electrospinning produces one-dimensional nanofiber morphologies (100–300 nm diameter) with excellent electronic percolation networks [102], but suffers from extremely low throughput and high equipment cost. The ball-milling–spray-drying coupled process combines the homogenization benefits of high-energy ball milling with the morphology control of spray drying. It generates uniform spherical secondary particles (5–10 μm) well-suited for electrode processing. A comprehensive comparison of all six methods is presented in Table 1.

### 2.4. Carbon Coating and Morphology Control

**Figure 3:**
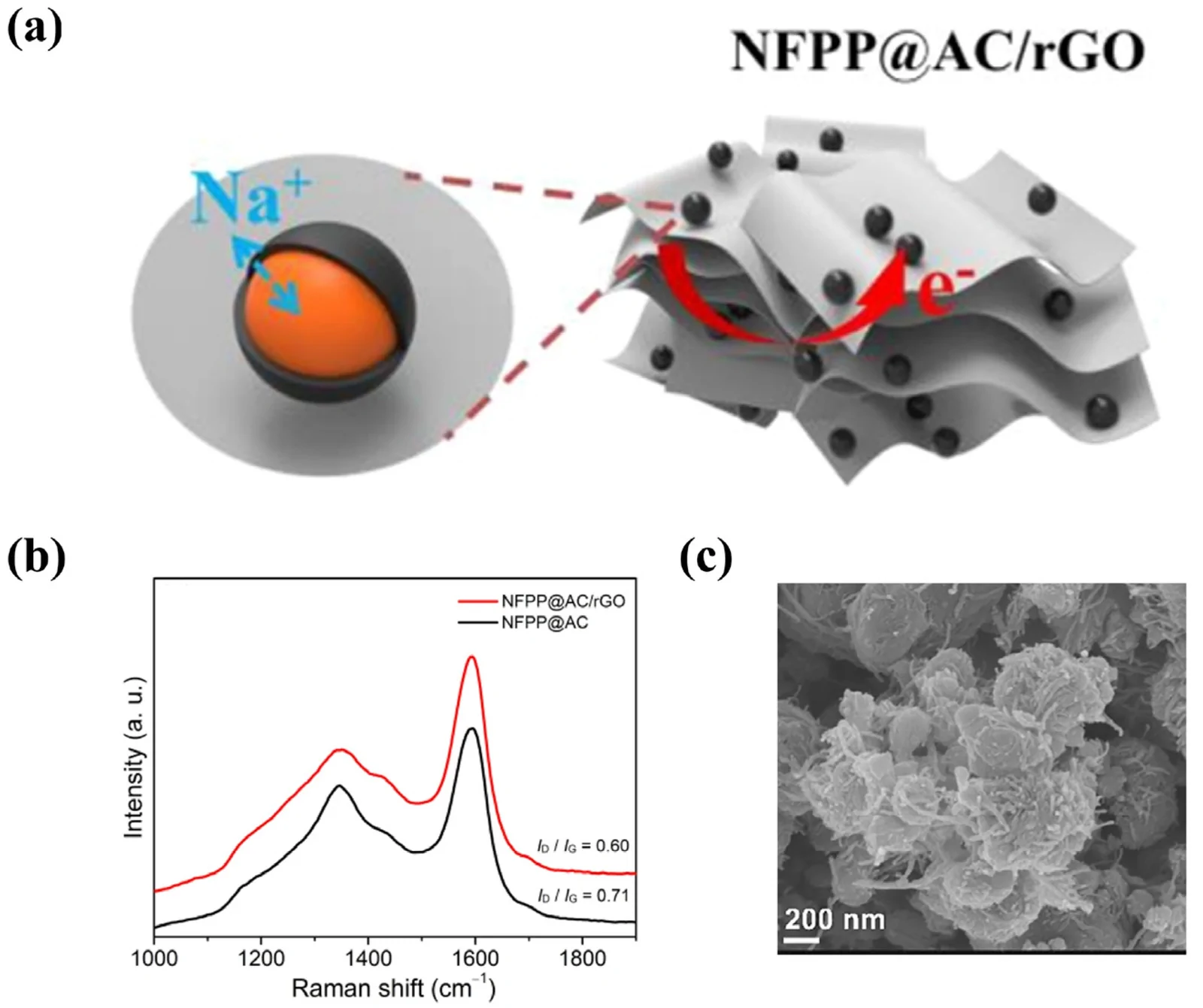
(**a**) Structural schematic of NFPP@AC/rGO; (**b**) Raman spectra of NFPP@AC/rGO and NFPP@AC [109]; (**c**) SEM image of NFMVPP/C@CNT [110].

Given the intrinsic electronic bandgap of NFPP (~2.99 eV), a conductive carbon coating is a prerequisite for practical electrode function. The carbon layer serves multiple roles: (i) providing electronic conduction pathways between NFPP particles and the current collector [103,104]; (ii) suppressing particle growth and agglomeration during high-temperature calcination [105]; (iii) moderating direct contact with the electrolyte while remaining ionically permeable, thereby preserving a continuous Na^+^ transport pathway [106]; and (iv) under certain conditions, participating in the formation of a favorable cathode–electrolyte interphase (CEI) [106,107]. The choice of carbon source strongly influences coating quality and morphology. Common precursors include monosaccharides (glucose, sucrose), organic acids (citric acid, ascorbic acid), polymers (polyethylene glycol PEG, polyvinylpyrrolidone PVP), pitch, and nanocarbon additives (reduced graphene oxide rGO, carbon nanotubes CNT). Raman spectroscopy assesses the degree of graphitization through the intensity ratio ID/IG of the D-band (~1350 cm^−1^, disordered carbon) to the G-band (~1580 cm^−1^, graphitic carbon); a lower ID/IG ratio signals higher electronic conductivity [108,109] (Figure 3b). High-resolution transmission electron microscopy (HRTEM) reveals carbon layer thicknesses typically in the 2–10 nm range. Conformal and uniform coatings, most reliably achieved through sol–gel and spray-drying routes, consistently deliver superior rate capability and cycling stability.

Designing a three-dimensional conductive network represents a critical advance beyond simple carbon coating. By incorporating CNTs (or rGO) as long-range conductive highways combined with an in situ pyrolytic amorphous carbon coating, a dual-carbon hierarchical conductive architecture can be constructed. Experiments confirm that this structure increases the specific surface area by approximately 78% and lowers the charge-transfer resistance by over 50%. Morphology control at the secondary-particle level is equally important. Spray-dried spherical particles offer optimal packing density and electrode processability. However, radial migration of the carbon precursor during droplet drying can produce an inhomogeneous carbon-depleted core/carbon-rich shell distribution. This heterogeneity drives non-uniform electrochemical reactions within individual particles. Optimizing process parameters, including slurry solid content, inlet/outlet temperature, and atomization pressure, is therefore essential to simultaneously provide the desired spherical morphology and uniform intra-particle carbon distribution [111]. Wei et al. constructed an NFPP-CNT hierarchical conductive network using CNTs and demonstrated excellent cycling stability at an ultra-high rate of 100C [112]. The carbon coating architecture and corresponding electrochemical performance are compared in Figure 3.

## 3. Phase Adjustment and Modification Strategies

The central point of this review is that phase-purity control represents the most fundamental materials science challenge facing NFPP. The diverse modification strategies developed over the past decade can each be understood as addressing this core challenge from a different angle. These include defect engineering, elemental doping, anion substitution, and heterostructure design. This section is organized along a progressive logic: problem (Phase Adjustment) to intrinsic regulation (defects/doping) to interfacial regulation (heterostructures). Each class of strategy is systematically analyzed in terms of mechanism and key literature.

### 3.1. Phase Purification: The Core Materials Challenge of NFPP

**Figure 4:**
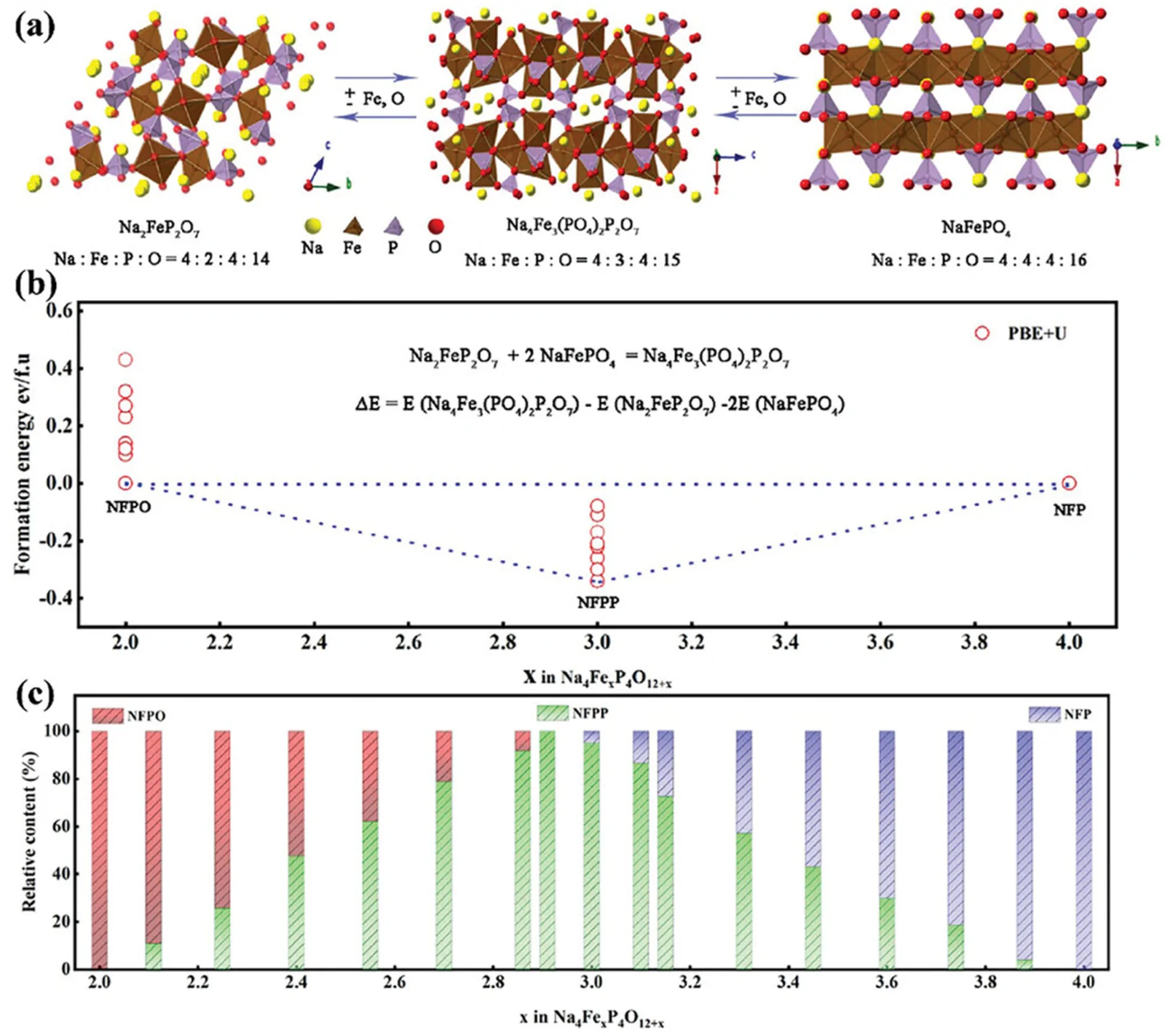
(**a**) Crystal structures of Na_2_FeP_2_O_7_,Na_4_Fe_3_(PO)_2_P_2_O_7_ and m−NaFePO_4_; (**b**) calculated formation energies of Na_2_FeP_2_O_7_,Na_4_Fe_3_(PO)_2_P_2_O_7_ and m−NaFePO_4_; (**c**) composition−dependent structural evolution in the Na_4+2x_Fe_3_(PO_4_)_2_P_2_O_7_ (2 ≤ x ≤ 4) system [45].

NFPP synthesis is fundamentally governed by a three-phase thermodynamic competition between the target phase Na_4_Fe_3_(PO_4_)_2_P_2_O_7_ (NFPP), maricite-type NaFePO_4_, and Na_2_FeP_2_O_7_. Of these competing phases, maricite-NaFePO_4_ is the most detrimental impurity (Figure 4a–c) [45,80,96]. Maricite is thermodynamically more stable than NFPP under typical synthesis conditions (550–700 °C). However, its dense close-packed structure entirely lacks the open Na^+^ diffusion channels characteristic of NFPP, rendering it electrochemically inert. Its presence dilutes the active material and lowers the gravimetric capacity [113]. More critically, it blocks Na^+^ transport pathways and disrupts the electronic percolation network, causing a disproportionately severe degradation of rate capability. Na_2_FeP_2_O_7_ (the pyrophosphate-rich phase) typically forms under sodium-excess synthesis conditions [114]. It exhibits limited electrochemical activity accompanied by sluggish kinetics. The coexistence of all three phases is not an incidental synthetic flaw. Rather, it reflects the narrow thermodynamic stability window of NFPP. Relative to the phase-separated products NaFePO_4_ + Na_2_FeP_2_O_7_, NFPP is in fact metastable [45]. It should be noted that when nanostructured or amorphized, maricite-NaFePO_4_ can exhibit appreciable reversible capacity; the detrimental role described here refers specifically to the dense, well-crystallized maricite that co-forms under NFPP synthesis conditions and blocks Na^+^ transport. The crystal structures and competitive relations among the three phases are shown in Figure 4.

Fan et al. demonstrated through a key mechanistic study that non-stoichiometric NFPP compositions fit within a “2NaFePO_4_·Na_2_FeP_2_O_7_” solid-solution framework. The Na:Fe ratio is the central compositional parameter governing phase purity [115]. Jablanovic et al. performed the first systematic Rietveld refinement-based quantitative phase analysis of the NFPP system [114]. They precisely determined the weight fractions of NFPP, maricite, and Na_2_FeP_2_O_7_. An optimal Na:Fe ratio of 4:2.9 yielded approximately 96 wt.% NFPP phase purity. Departures from this optimum led to rapid impurity accumulation: maricite increased under Na-deficient conditions and Na_2_FeP_2_O_7_ increased under Na-rich ones. This quantitative composition–performance correlation marks the pivotal transition from empirical trial-and-error to rational compositional design in NFPP research.

**Figure 5:**
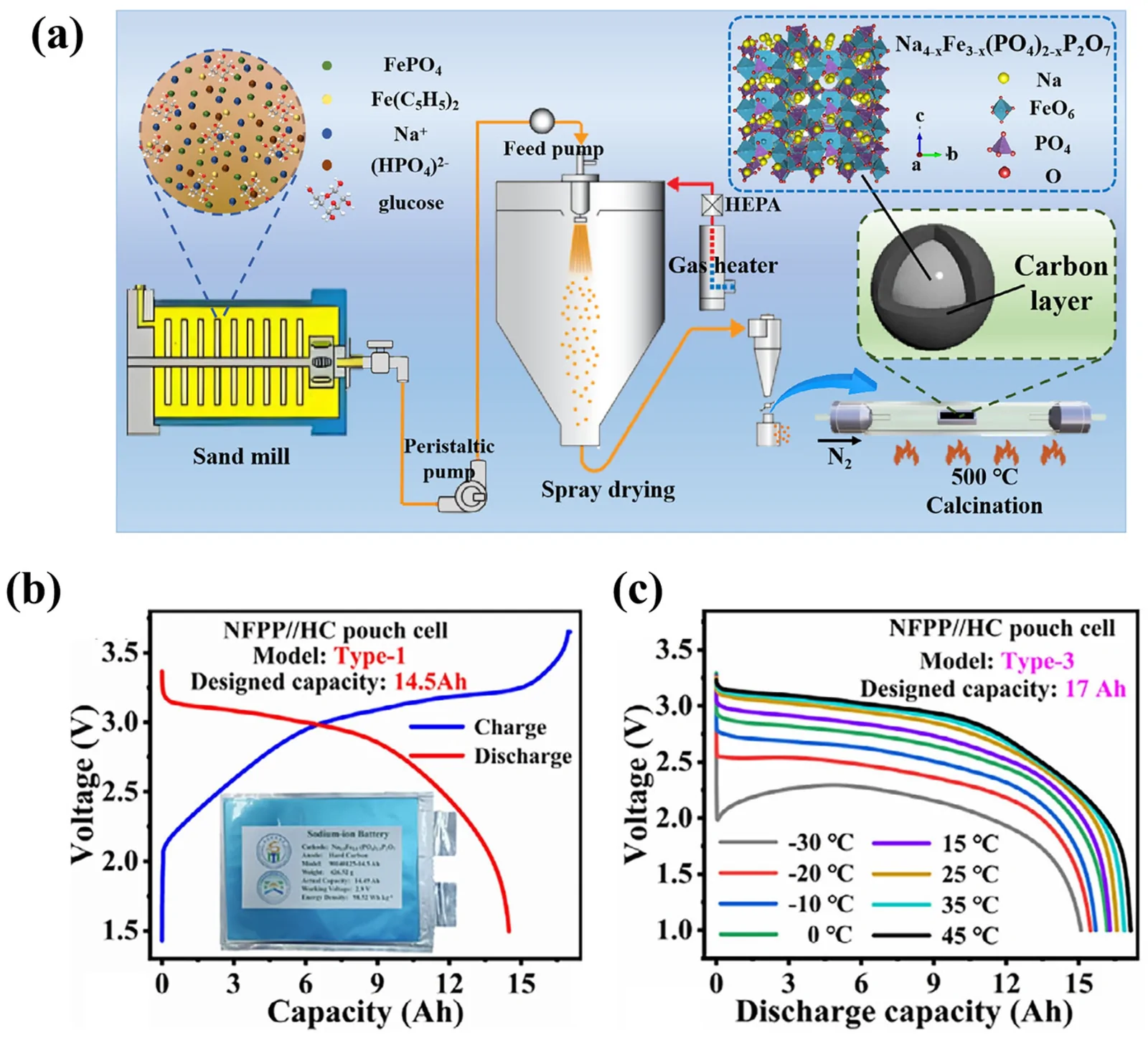
(**a**) Schematic of the dual-iron-source and non-stoichiometric strategy for the synthesis of Na_4−x_Fe_3−x_(PO_4_)_2−x_P_2_O_7_ materials; (**b**) initial charge/discharge curves of Na_3.5_Fe_2.5_PP//HC pouch cells; (**c**) wide-temperature-range performance of Na_3.5_Fe_2.5_PP//HC pouch cells [26].

Three major strategies have been developed to address the phase-purity challenge. The first—non-stoichiometric tuning—exploits the sensitivity of phase equilibria to the Na:Fe ratio. Systematic mapping of phase-purity windows against composition and synthesis temperature revealed that Na:Fe ratios in the range of 4:2.85–4:2.95, combined with calcination at 550–650 °C, yield the highest NFPP phase fraction [114]. The second strategy is dual-iron-source engineering. It replaces conventional single-iron-source precursors with a carefully designed pair, such as FePO_4_ + FeC_2_O_4_·2H_2_O. Their complementary reaction pathways preferentially promote NFPP nucleation while suppressing maricite formation [68]. Specifically, one iron source reacts earlier and serves as a P-rich intermediate template, while the other releases Fe more gradually; their staggered, complementary decomposition maintains a locally optimal Na:Fe:P ratio throughout the heat treatment, which preferentially nucleates NFPP and kinetically suppresses the maricite-NaFePO_4_ that would otherwise arise from local Fe/P fluctuations. Wang et al. validated this approach at the kilogram scale. Their assembled 13 Ah pouch cell maintained stable capacity over 1000 cycles—a critical demonstration of industrial feasibility. The third strategy uses self-sacrificial precursors. Deliberately designed intermediate phases—such as amorphous FePO_4_·xH_2_O or Na-rich sacrificial intermediates—decompose during calcination in a time-controlled manner. This releases active species along a pathway that steers the reaction away from maricite and toward NFPP crystallization. Chen et al. reported that this self-sacrificial amorphous precursor-mediated route yielded 99.75% capacity retention after 2000 cycles, attributed to near-complete suppression of maricite impurities [116]. Figure 5 shows the detailed reaction pathway of dual-iron-source engineering.

### 3.2. Defect Engineering

#### 3.2.1. Iron Vacancy Engineering

**Figure 6:**
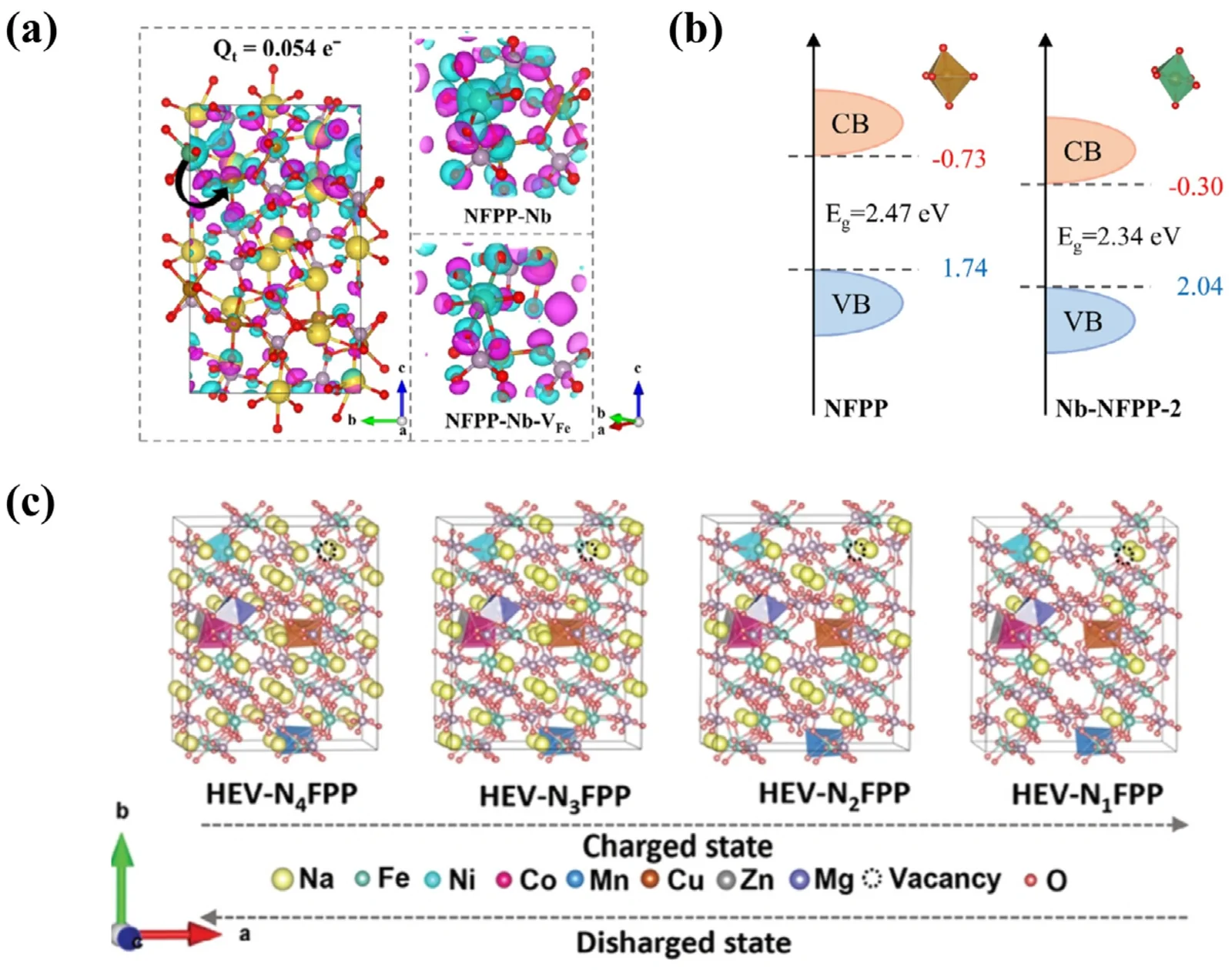
(**a**) Differential charge density and (**b**) energy band diagrams of NFPP and Nb−NFPP−2 [35] (CB and VB represent the conduction band and valence band, respectively); (**c**) the optimized crystal structure of HEV−NFPP and its evolution during the sodiation/desodiation processes [67].

The intentional introduction of Fe vacancies, expressed as Na_4_Fe_3−x_(PO_4_)_2_P_2_O_7_, has emerged as a dual-purpose strategy. It simultaneously addresses two performance limitations of NFPP: sluggish Na^+^ diffusion kinetics and underutilized theoretical capacity [117]. DFT calculations reveal that Fe vacancies induce local lattice distortions that expand the bottleneck cross-sectional area of adjacent Na^+^ migration channels [35,118,119,120]. Concurrently, the charge compensation accompanying Fe^2+^ removal generates additional electrostatically favorable Na^+^ sites, effectively increasing the available storage capacity and boosting practical specific capacity (Figure 6a,b). Notably, combining Fe vacancies with a high-entropy concept represents the state-of-the-art in NFPP cycle life. Hu et al. achieved over 15,000 ultra-stable cycles through synergistic high-entropy doping and Fe vacancy design. A critical distinction is essential: unintentional Fe vacancies from imperfect synthesis generally impair performance by disrupting the local structure (Figure 6c) [67]. In contrast, deliberately engineered Fe vacancies, introduced through precise stoichiometric control, simultaneously optimize ion transport and capacity.

#### 3.2.2. Sodium Vacancy Engineering

**Figure 7:**
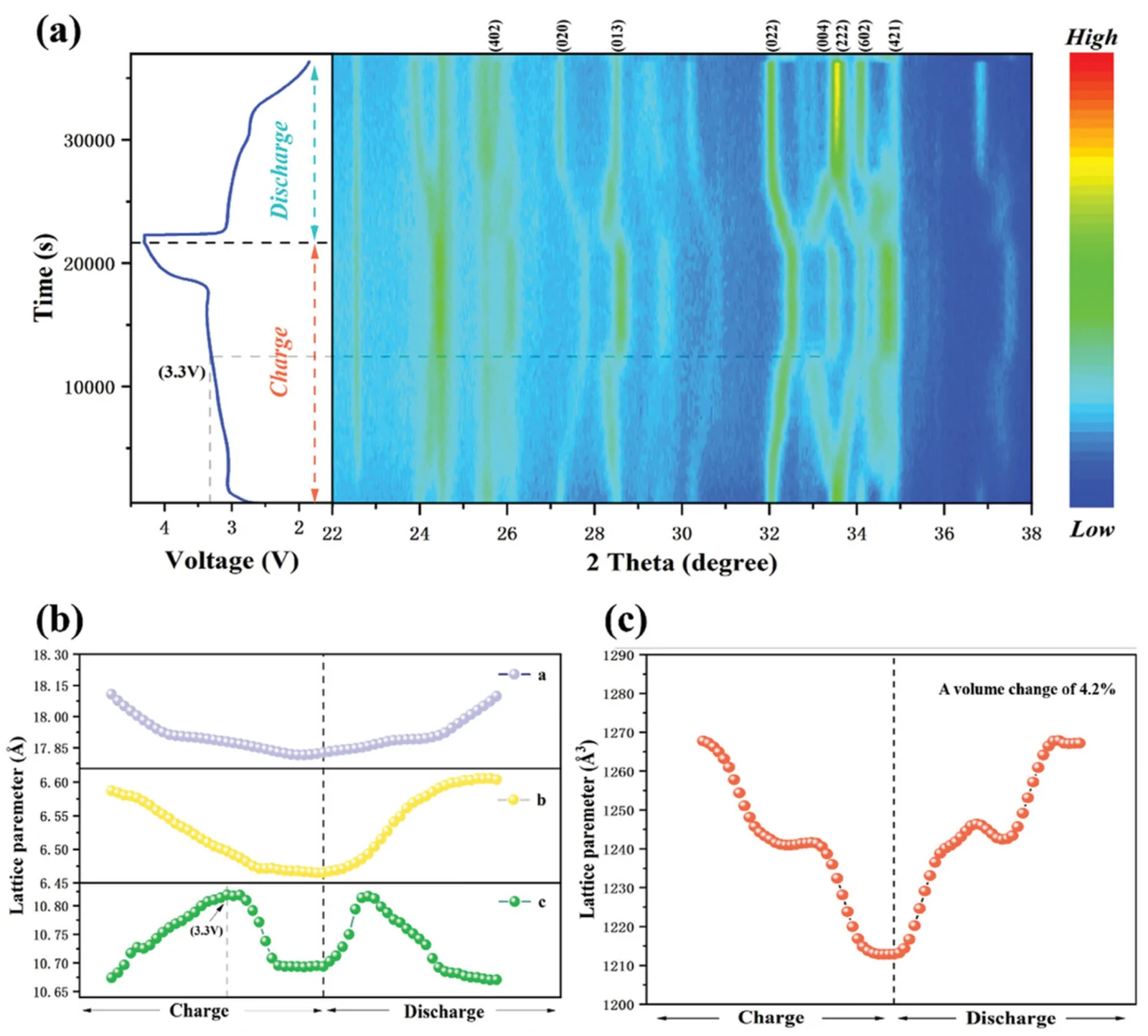
(**a**) In situ XRD patterns collected during the first cycle of the NFPP-La^3+^ electrode at 0.2C between 1.7 and 4.3 V; (**b**,**c**) changes in cell parameters and volume produced by Na^+^ extraction/insertion [117].

Where Fe vacancies subtract, Na vacancies pre-empty. Na vacancy engineering removes Na^+^ from the as-synthesized structure to pre-create vacant sites that increase the Na^+^ chemical diffusion coefficient [117]. Chen et al. targeted Na^+^ diffusion kinetics through Na-site vacancy engineering. They introduced Na vacancies by doping La^3+^ at Na sites, producing Na_4−3x_La_x_□_2x_Fe_3_(PO_4_)_2_(P_2_O_7_)/C. The charge compensation mechanism—each La^3+^ dopant generates two VNa—widens Na^+^ transport channels, while the pillar effect of La^3+^ mitigates lattice stress during long-term cycling (Figure 7a–c). The optimized sample, Na_3.91_La_0.03_□_0.06_Fe_3_(PO_4_)_2_(P_2_O_7_)/C, retained 99.45 mAh g^−1^ at 20C and 92.36% capacity after 1000 cycles at 10C. This work opens a new avenue for high-rate, long-cycle-life NFPP cathode design through Na-site vacancy engineering. In situ XRD characterization and theoretical calculations are presented in Figure 7. The first-cycle irreversible capacity of the La^3+^-doped NFPP originates mainly from (i) irreversible Na^+^ trapping in the newly activated Na sites during initial activation and (ii) electrolyte decomposition/CEI formation on the fresh carbon and electrode surfaces; the slight irreversible lattice rearrangement observed in the in situ XRD (Figure 7a) as the La^3+^ “pillar” sites establish their equilibrium occupancy is consistent with a fraction of Na^+^ not being fully recovered on the first return, a loss that is confined to the first cycle and does not recur thereafter.

#### 3.2.3. Oxygen Vacancy Regulation

**Figure 8:**
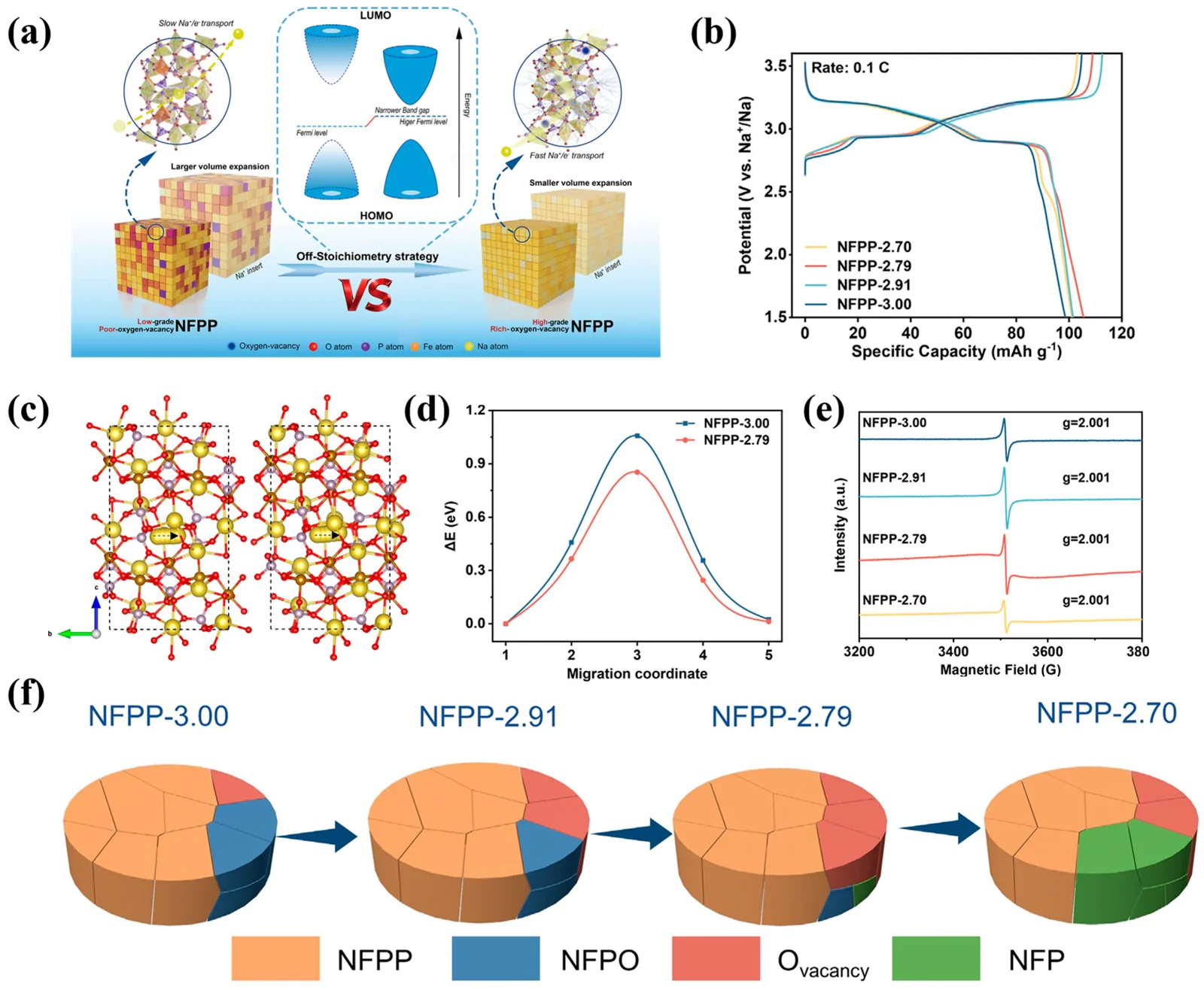
(**a**) Design strategy toward high-performance NFPP materials with abundant oxygen vacancies. (**b**) GCD curves of NFxPP cathodes at 0.1 C. Comparison of (**c**) diffusion pathways and (**d**) corresponding diffusion energy barriers in NFPP and Na_4_Fe_2.79_(PO_4_)_2_P_2_O_7_ frameworks. (**e**) EPR plots of different samples. (**f**) Illustration of the phase transformation of the NFPP/m−NFP/NFPO system [54].

Oxygen vacancy engineering stands among the most exciting breakthroughs in NFPP research during 2025–2026. The Xiao group conducted systematic studies on tuning the oxygen vacancy concentration in NFPP. By precise controlling the reducing atmosphere during calcination, Ran et al. reached tunable oxygen vacancy concentrations [54]. The optimal composition (NFPP-2.79, denoting the O stoichiometric ratio) delivered threefold performance enhancement (Figure 8a): (i) oxygen vacancies introduce shallow donor levels within the band gap, effectively narrowing the electronic band gap and boosting electronic conductivity; (ii) Na–O bond strength weakens near oxygen vacancies, lowering the Na^+^ diffusion barrier from ~0.53 to ~0.38 eV; and most critically, (iii) the vacancy-rich NFPP-2.79 was successfully scaled to Ah-level pouch cells, retaining 75.63% capacity after 3000 cycles and passing a stringent nail-penetration safety test without thermal runaway (Figure 8b–f). Gao et al. independently demonstrated that F/Si co-doping indirectly generates and stabilizes oxygen vacancies, compressing the unit-cell volume change during charge–discharge from ~4% to merely 1.5%—approaching the ideal of a perfect “zero-strain” cathode [63]. A key insight from these studies is the “volcano-type” relation between oxygen vacancy concentration and electrochemical performance: insufficient concentration yields limited improvement, while excessive concentration triggers irreversible structural collapse—underscoring the importance of precise defect concentration control. Figure 8 shows the systematic correlation between oxygen vacancy regulation strategies and electrochemical performance.

### 3.3. Elemental Doping Strategies

#### 3.3.1. Alkali-Metal Doping at Na Sites

Substitutional doping at Na sites with smaller alkali cations (primarily Li^+^) or larger cations (K^+^) draws on the “ion pillar” concept proposed for layered oxide cathodes. Li^+^ (ionic radius 0.76 Å) is smaller than Na^+^ (1.02 Å) and preferentially occupies the Na3 site—a site that remains partially occupied during cycling and serves as a structural pillar [121,122,123]. The stronger Li–O bonds (relative to Na–O) reinforce the local framework, suppressing channel collapse during deep Na^+^ extraction and thereby unlocking capacity at higher cutoff voltages. Conversely, K^+^ doping (ionic radius 1.38 Å) expands the lattice spacing and widens Na^+^ migration channels, lowering the diffusion barrier. DFT-calculated doping formation energies indicate that Li^+^ preferentially occupies the Na3 site, whereas K^+^ favors the Na1 site due to its larger coordination volume [122]. Li-doped NFPP (Na_4−x_LixFe_3_(PO_4_)_2_P_2_O_7_) reportedly delivers a reversible capacity of 110.6 mAh g^−1^ at 0.05C, though the reversibility of Li^+^ de/intercalation and the long-term stability of Li occupancy at the Na3 site require further confirmation [40]. Because K^+^ (1.38 Å) is much larger than Na^+^ (1.02 Å), its substitution expands the lattice and widens the Na^+^ migration channels, which DFT predicts lowers the Na^+^ diffusion barrier (K^+^ preferentially occupies the larger Na1 site); however, the larger radius also introduces local lattice strain and, at excessive doping levels, can destabilize the framework and slightly reduce the theoretical capacity, since K^+^ is electrochemically inactive in a Na cell. K^+^ doping is therefore expected to improve rate capability and structural stability up to an optimal low concentration, beyond which lattice strain and capacity dilution become limiting.

#### 3.3.2. High-Valence Cation Doping at Fe Sites

(a) Vanadium Doping

**Figure 9:**
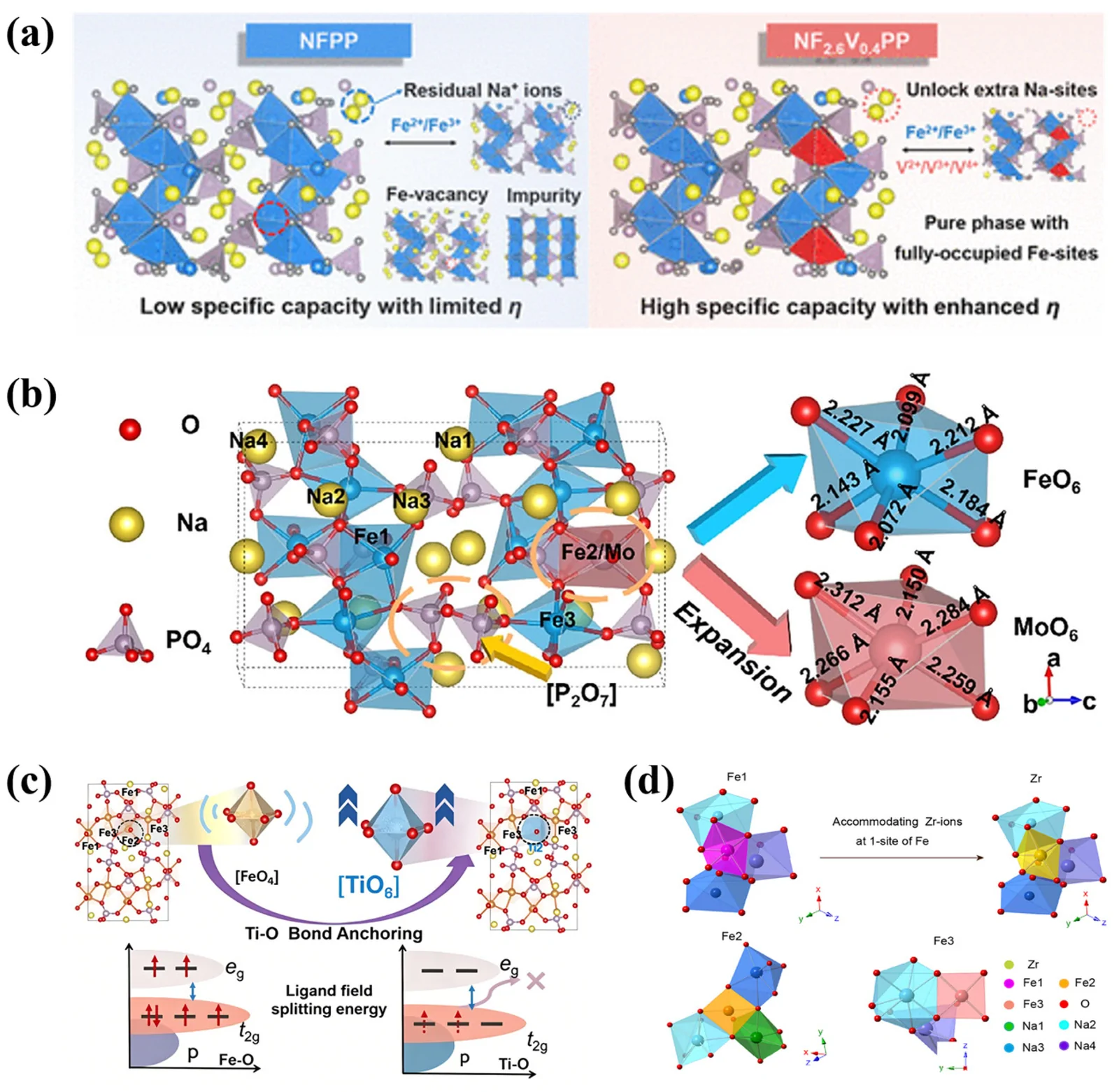
(**a**) Schematic of the V-doping mechanism in NFPP [124]; (**b**) the crystal structure of NFPP-2Mo and the bond length comparison of (Mo and Fe) [126]; (**c**) schematic of the effect of Ti doping on NFPP structure [129]; (**d**) local schematic of Zr doping at different Fe sites in NFPP [36].

V^3+^ doping is the earliest investigated and currently the most mature and productive doping strategy for the NFPP system. Compared with Fe^2+^ (0.78 Å), the smaller ionic radius of V^3+^ (0.64 Å) induces local lattice contraction, compressing M–O bond lengths and enhancing structural rigidity (Figure 9a). Crucially, V^3+^/V^4+^ is electrochemically active within the NFPP voltage window, providing an additional redox couple at ~3.4 V (vs. Na^+^/Na) that contributes supplementary capacity to the Fe^2+^/Fe^3+^ redox pair [124,125]. Zhang et al. reported that Na_4−x_Fe_3−x_V_x_(PO_4_)_2_P_2_O_7_ delivered 123.4 mAh g^−1^ at 0.1C and 81.65% capacity retention after 10,000 cycles at 20C [90]. The field reached a new milestone in 2026: Li et al. published a “V^3+^ coordination engineering” strategy in *Nature Energy*, activating originally electrochemically inert Na sites through precise tuning of the V^3+^ coordination environment [66]. This approach yielded a record capacity of 150.7 mAh g^−1^ and an energy density of 487 Wh kg^−1^, surpassing what was previously considered the performance ceiling of the NFPP system.

(b) Aluminum Doping and V/Al Co-Substitution

Al^3+^ doping carries rich structural-reference significance: since Al^3+^ has no accessible redox couple within the electrolyte stability window, any performance gains from Al doping can be unambiguously attributed to structural stabilization rather than additional redox capacity. The Al–O bond dissociation energy (512 kJ mol^−1^) is substantially higher than that of Fe–O (409 kJ mol^−1^), quantitatively accounting for the enhanced framework rigidity conferred by Al^3+^ substitution [55]. Zhao et al. recently reported a V^3+^/Al^3+^ co-substitution strategy that leverages the complementary advantages of both dopants: V^3+^ provides active redox capacity and electronic conductivity enhancement, while Al^3+^ reinforces structural stability. The synergy exceeds additivity: the co-substituted material sees its electronic band gap change from 2.99 (pristine NFPP) to 1.53 eV, and the Na^+^ diffusion barrier drop from 0.529 to 0.398 eV (Figure 9c). This nonlinear enhancement embodies the “doping synergy” design principle—combining elements with complementary functions (redox activity + structural stabilization) yields performance that surpasses the sum of their individual contributions.

(c) Mo^6+^ Doping—An Orbital Delocalization Strategy

Mo^6+^ doping represents a conceptual breakthrough that departs from the structural stabilization paradigm. Jian et al. proposed an “orbital delocalization-assisted valence regulation” strategy: extended 4d orbitals of molybdenum hybridize with O 2p states to form a delocalized electronic band, closing the band gap and dramatically enhancing electronic conductivity [126]. The Mo^6+^/Mo^4+^ redox couple operates at ~3.0 V (vs. Na^+^/Na), providing additional charge compensation capacity. Unexpectedly, Mo doping was found to electrochemically activate the Na2 site—largely inert in pristine NFPP—further increasing the available storage capacity (Figure 9b). Mo-NFPP is characterized by a record capacity of 130.74 mAh g^−1^ at 0.1C, along with outstanding rate capability (87.23% retention after 10,000 cycles at 50C) and wide-temperature operability (−40 °C to +60 °C). A key mechanistic question raised by this work is: how can the highly charged Mo^6+^—with its strong electrostatic repulsion—be energetically feasible at Fe^2+^ sites? Jian et al. demonstrated through DFT calculations that the energy penalty of Mo^6+^ incorporation is compensated by the electronic energy gain from orbital hybridization and the electrostatic screening provided by the polarizable polyanion framework—offering a quantitative theoretical rationale for this seemingly counterintuitive doping strategy.

(d) Ti^4+^/Zr^4+^ Doping

Ti^4+^ and Zr^4+^ doping in NFPP have received relatively limited attention, yet they offer distinct advantages. Ti^4+^ (0.605 Å) can induce a strong electron–lattice coupling effect, already validated in the NaMnPO4 system and potentially applicable to NFPP [127,128,129]. Zr^4+^ (0.72 Å), with its larger ionic radius, expands the lattice spacing and widens Na^+^ diffusion channels (Figure 9d) [36]. The high charge density of Ti^4+^ and Zr^4+^ also helps stabilize the surrounding oxygen coordination environment, which may suppress the Fe dissolution processes responsible for long-term capacity fade (Figure 9c) [129]. The modest body of work on Ti/Zr doping in NFPP points to a promising avenue for future exploration, especially given the successful precedent of Ti doping in related polyanion cathode systems, where dopants of different radii act in complementary fashion.

(e) Nb^5+^ doping.

Nb^5+^ doping is a novel high-valence strategy reported in 2026. Hou et al. found that heterovalent substitution of Nb^5+^ (0.64 Å) at Fe^2+^ sites induces Fe vacancy formation through a charge compensation mechanism, achieving two effects at once: hybridization of Nb 4d orbitals with O 2p states narrows the electronic bandgap, while the attendant Fe vacancies widen Na^+^ diffusion channels [35]. Charge density difference analysis further revealed that the enhanced covalent character of Nb–O bonds plays a critical role in stabilizing the crystal structure during cycling.

High-valence cation doping at Fe^2+^ sites—whether redox-active (V^3+^, Mo^6+^) or electrochemically inert (Al^3+^, Ti^4+^, Zr^4+^, Nb^5+^)—confers several convergent benefits on NFPP. Structurally, the stronger M–O bonds relative to Fe–O contract and rigidify the local lattice, suppressing maricite-NaFePO_4_ formation and enhancing long-term cycling stability. Electronically, the extended d-orbitals of high-valence dopants hybridize with O 2p states, narrowing the bandgap and improving intrinsic electronic conductivity. Kinetically, the charge compensation mechanisms (Fe^2+^→Fe^3+^ oxidation or Fe vacancy creation) generate beneficial defects that lower the Na^+^ diffusion barrier. However, these advantages come with inherent trade-offs: electrochemically inert dopants contribute no additional redox capacity and dilute the energy density when used in excess; charge-compensation-induced Fe vacancies, while kinetically beneficial, inevitably reduce the number of active Fe redox centers; and redox-active dopants such as V may introduce additional voltage plateaus that complicate battery management. A rational design guideline is therefore that redox-active high-valence dopants (V, Mo) are best suited for simultaneously boosting capacity and rate capability, whereas structurally stabilizing yet electrochemically inert dopants (Al, Ti, Zr) should be employed at minimal concentrations—or co-doped with redox-active elements—to avoid sacrificing energy density. The emerging principle of synergistic co-doping (e.g., V/Al, Ti/Al, high-entropy combinations) leverages the complementary strengths of different dopants while mitigating their individual drawbacks, representing the most promising direction for future NFPP cathode design.

#### 3.3.3. Low-Valence Transition Metal Doping (Mn^2+^, Ni^2+^)

**Figure 10:**
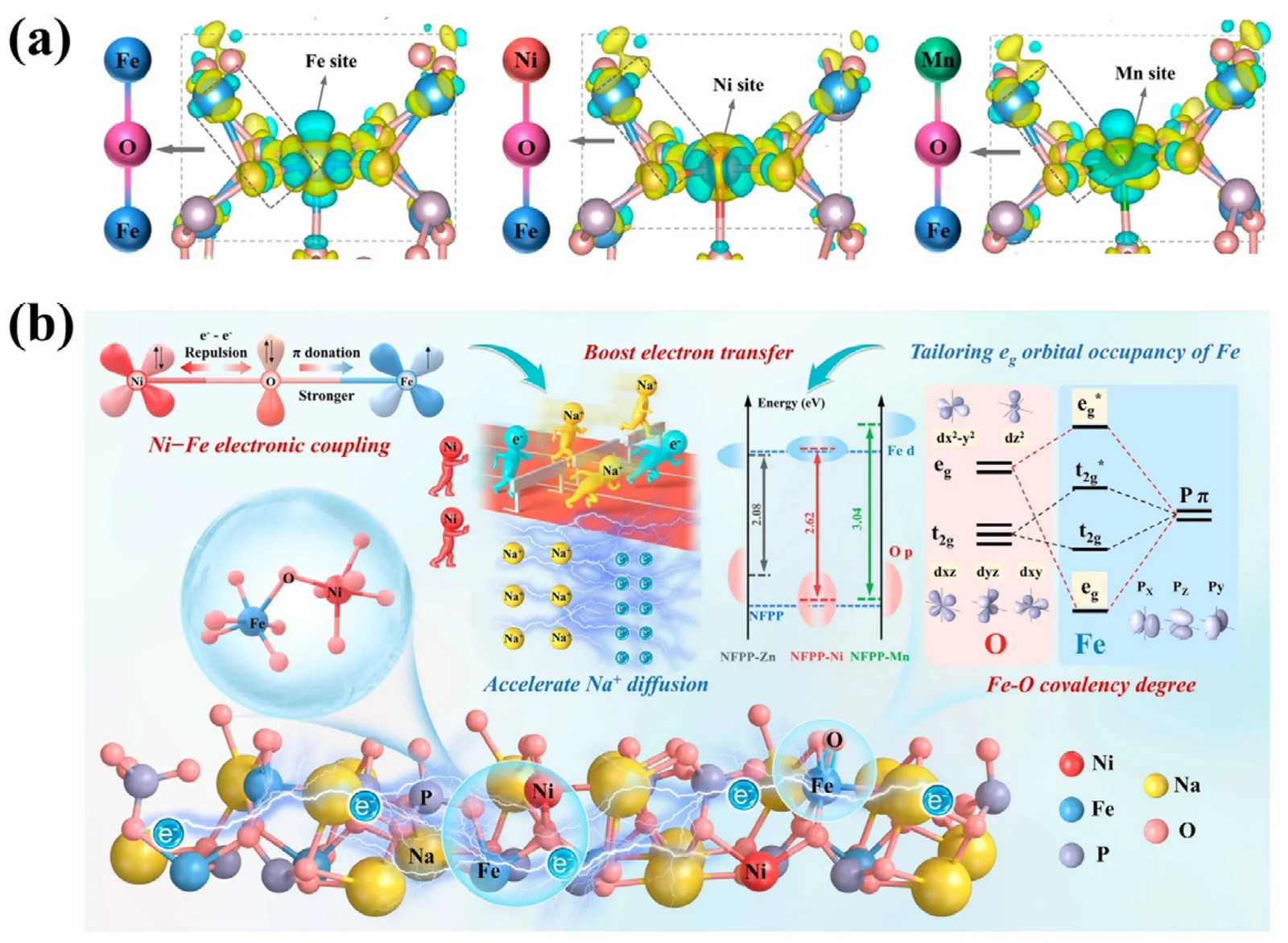
(**a**) Difference charge densities of NFPP, NFPP−Ni, and NFPP−Mn; (**b**) Schematic illustration of Ni–Fe electronic coupling and changes in eg orbital occupancy in the NFPP cathode following Ni doping (* denotes an antibonding orbital) [20].

Unlike the high-valence cation doping mechanisms discussed above, Mn^2+^ and Ni^2+^ doping operates primarily by suppressing the formation of the maricite impurity phase. Subaşi et al. employed a combination of advanced characterization techniques—X-ray absorption spectroscopy (XAS) and resonant inelastic X-ray scattering (RIXS)—to elucidate that substitution of Mn^2+^ and Ni^2+^ at Fe^2+^ sites alters the local chemical potential, rendering the target NFPP phase thermodynamically more stable and thereby inhibiting maricite nucleation [56]. Mn-NFPP delivers approximately 92 mAh g^−1^ at 0.1C with 99.5% capacity retention after 100 cycles, prioritizing cycling durability. Ni-NFPP, by contrast, benefits from the Ni^2+^/Ni^4+^ redox couple operating at a high voltage plateau of approximately 4.1 V, yielding higher energy density, though the elevated voltage poses challenges for electrolyte stability. A comprehensive cross-comparison across the Fe–Mn–Ni–Co NFPP composition space reveals the intrinsic trade-offs among capacity, voltage, stability, and cost, providing a rational framework for application-specific composition optimization. Wang et al. further elucidated the electronic structure origin of Ni-doping benefits through a combined DFT and experimental study [118]. They obtained that Ni^2+^ substitution at Fe^2+^ sites effectively tailors the eg orbital occupancy of Fe, strengthening Ni–Fe electronic coupling and optimizing the local electronic configuration, thereby enhancing electronic conductivity and reducing the Na^+^ migration barrier (Figure 10a,b). Charge density difference analysis of Ni and Mn doping is presented in Figure 10.

In contrast to the high-valence doping mechanisms discussed above, low-valence transition metal doping (Mn^2+^, Ni^2+^) operates through a fundamentally different paradigm. As isovalent substituents for Fe^2+^, Mn^2+^ and Ni^2+^ do not rely on charge-compensation-induced defect generation; instead, they stabilize the target NFPP phase primarily through thermodynamic modulation of phase equilibria, effectively suppressing maricite-NaFePO_4_ formation without introducing Fe vacancies. Mn^2+^ doping offers the additional advantage of a Mn^2+^/Mn^3+^ redox couple at elevated potentials (~3.5 V vs. Na^+^/Na), contributing supplementary capacity beyond the Fe^2+^/Fe^3+^ plateau. Ni^2+^ doping, on the other hand, modulates the eg orbital occupancy of Fe, enhancing Fe–O bond covalency and improving electronic conductivity through Ni–Fe electronic coupling. Nevertheless, low-valence doping is not without limitations: the Jahn–Teller distortion associated with Mn^3+^ can compromise long-term structural integrity, particularly at high degrees of desodiation. Ni^2+^, being electrochemically inactive within the NFPP operating voltage window, reduces the theoretical specific capacity when introduced in large quantities; and the absence of charge-compensation-induced vacancies means that Na^+^ diffusion enhancement is less pronounced than in high-valence-doped systems. The choice between high-valence and low-valence dopants should therefore be guided by the primary performance target: high-valence dopants are preferred when rate capability and structural rigidity are prioritized, whereas low-valence dopants—particularly Mn^2+^—are advantageous when phase purity and supplementary high-voltage capacity are the principal objectives.

#### 3.3.4. High-Entropy Doping: Multi-Element Synergistic Effects

**Figure 11:**
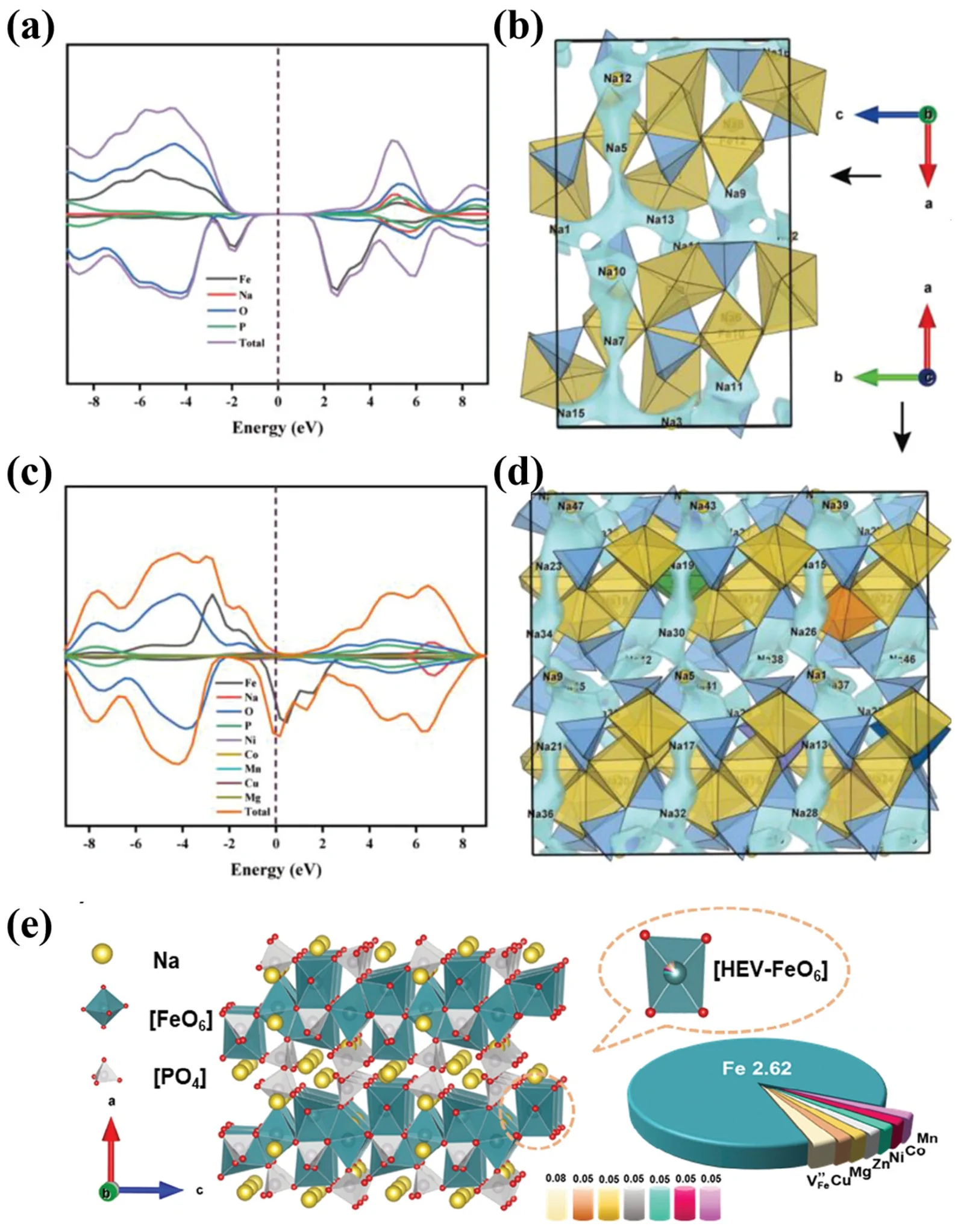
The density of states (DOS) of (**a**) NFPP and (**c**) NFPP−HE. The bond valence energy landscapes (BVEL) and Na^+^ migration pathways of (**b**) NFPP and (**d**) NFPP−HE [130]. (**e**) Schematic representation of the NFPP crystal structure and component design [67].

High-entropy doping represents a paradigm shift in NFPP materials design, moving from single-element to multi-element compositional engineering. By introducing multiple dopant elements (typically ≥5) in near-equimolar proportions at Fe sites, the configurational entropy (ΔS_config = −R∑xilnxi) is maximized, lowering the Gibbs free energy of the NFPP phase and preferentially stabilizing it over the competing maricite phase [130]. Ge et al. reported the first high-entropy NFPP cathode—Na_4_Fe_2.885_(NiCoMnCuMg)_0.023_(PO_4_)_2_P_2_O_7_—achieving 122 mAh g^−1^ at 0.1C and retaining 82.3% of that capacity at 50C. The high-entropy strategy harnesses three synergistic effects: (i) entropy stabilization—a high ΔS_config lowers the total free energy and broadens the NFPP phase stability window; (ii) the cocktail effect—each dopant contributes a distinct function: Mg widens diffusion channels, Cu enhances electronic conductivity through its 3d^10^ electron configuration, and Mn/Ni/Co supply additional redox capacity (Figure 11a–d); (iii) the lattice distortion effect—the spatial distribution of ions with different radii generates heterogeneous local strain fields, which can be directionally tuned to optimize Na^+^ migration pathways (Figure 11e). Hu et al. combined high-entropy doping with Fe vacancy engineering in a synergistic design, achieving the highest long-term cycling stability reported to date for NFPP, exceeding 15,000 cycles [67]. DFT calculations of the electronic structure and ion transport properties of high-entropy-doped NFPP are presented in Figure 11.

### 3.4. Anion Substitution

**Figure 12:**
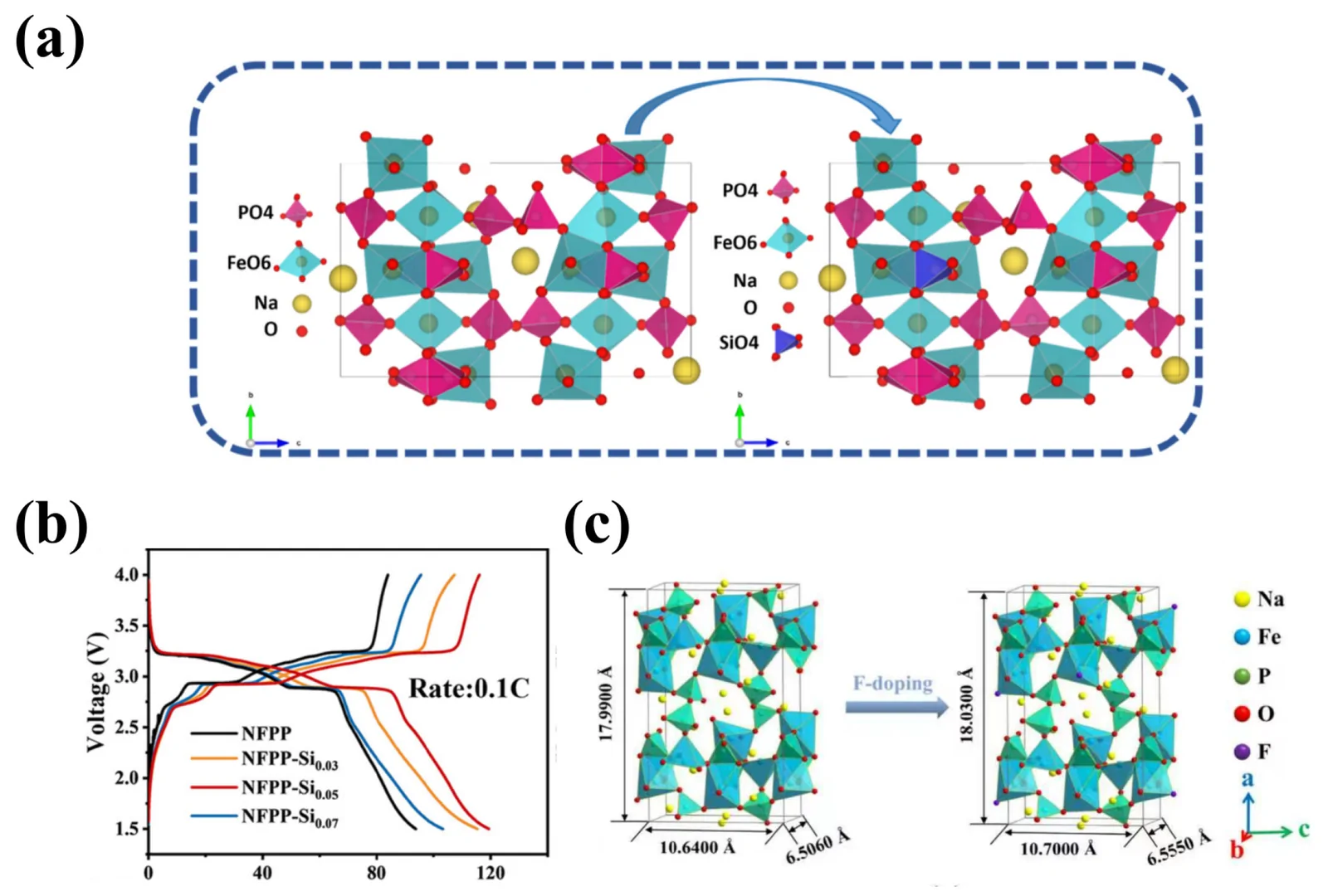
(**a**) Schematic of lattice changes upon SiO_4_^4−^ substitution for PO4^3−^; (**b**) charge/discharge curves at 0.1 C of NFPP and NFPP-Six (x = 0.03, 0.05 and 0.07) [62]; (**c**) schematic of lattice changes upon F^−^ interstitial incorporation [132].

Cation doping acts on the Fe–O octahedral units, whereas anion substitution directly targets the polyanion framework (PO_4_^3−^/P_2_O_7_^4−^) that constitutes the structural backbone of NFPP, thereby altering the fundamental electronic and structural properties at a deeper level [131]. Liu et al. pioneered the use of SiO_4_^4−^ to replace PO_4_^3−^ [62]. DFT calculations showed that SiO_4_^4−^ preferentially occupies the P1 site, which has the lowest formation energy, expanding the a-axis cell parameter and enhancing Na^+^ diffusion kinetics (Figure 12a,b). Si-NFPP delivers 119.4 mAh g^−1^ at 0.1C with 84.2% capacity retention after 5000 cycles at 50C. The charge mismatch between SiO_4_^4−^ (Si^4+^) and PO_4_^3−^ (P^5+^) must be compensated by fine-tuning the Na^+^ stoichiometry—an aspect that merits careful quantitative analysis in future studies.

F^−^ doping offers two incorporation modes with distinctly different consequences. Cao et al. showed through DFT calculations that F^−^ can substitute O^2−^ sites or occupy interstitial positions in the NFPP lattice. The interstitial mode is energetically more favorable. The voltage shift has been attributed to the strong electron-withdrawing character of F^−^, which increases the ionicity of Fe–O bonds and thereby raises the Fe^2+^/Fe^3+^ redox potential (Figure 12b) [132]. Gao et al. reported an F/Si co-doping strategy that simultaneously generates and stabilizes oxygen vacancies. The synergistic mechanism proceeds through three coupled steps: Si^4+^ substitution for P^5+^ alters the framework charge distribution; F^−^ interstitial incorporation enhances local polarization; and together they promote oxygen vacancy formation (Figure 12c) [63]. The resulting material exhibits a unit-cell volume change of only 1.5% during charge and discharge, approaching the ideal of a truly zero-strain cathode. Despite these impressive experimental achievements, theoretical studies of anion doping in NFPP lag substantially behind. Systematic DFT investigations are urgently needed to map dopant site preference, electronic structure evolution, and Na^+^ diffusion energetics as functions of anion dopant type and concentration. The lattice modulation effects of various anion substitution strategies are shown in Figure 12.

### 3.5. Heterostructures and Dual-Phase Design

**Figure 13:**
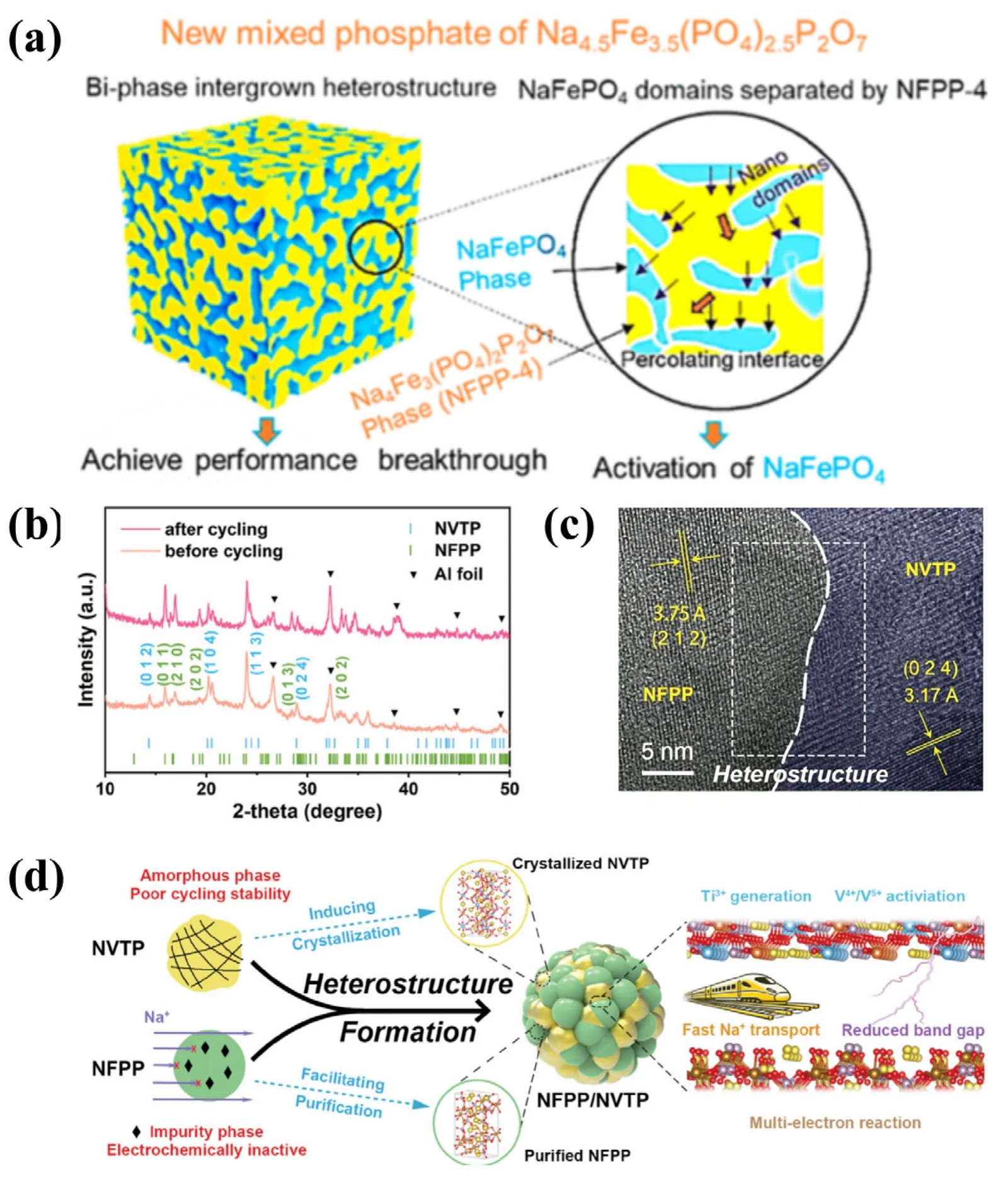
(**a**) Schematic image of the NFPP-4.5 cathode with a two-phase intergrown heterostructure of NFP and NFPP-4 [64]; (**b**) XRD pattern and (**c**) HRTEM image of the NFPP/NVTP electrode after long-term cycling; (**d**) schematic of synergistic effects in NFPP/NVTP [133].

The strategies discussed so far share a common goal: obtaining or approaching single-phase NFPP products. Heterostructure design represents a conceptual inversion of this paradigm. Instead of eliminating secondary phases, one actively designs multiphase composites whose interphase boundaries deliver functional advantages beyond those of any single phase. Xu et al. demonstrated a symbiotic heterostructure between NFPP and Na_4_Fe_3_(PO_4_)_2.5_(P_2_O_7_), a phosphate-rich variant designated as NFP (Figure 13a) [64]. In this intergrown architecture, the NFP phase supplies higher Fe content and thus higher capacity, while the NFPP phase provides the structural framework that constrains volume change. Crucially, the two phases intergrow at the atomic scale rather than mixing physically at the particle level. This enables a “nonlinear superposition” of properties, yielding an energy density of approximately 400 Wh kg^−1^.

La et al. explored a complementary heterostructure strategy by combining NFPP with the NASICON-type fast-ion conductor Na_3_V_2_(PO_4_)_3_ (NVP). They found that spontaneous charge redistribution occurs at the NFPP/NVP heterointerface, generating a built-in electric field that facilitates Na^+^ transport across the phase boundary. This constitutes a “1 + 1 > 2” interfacial enhancement effect. The NFPP–NaFePO_4_–Na_2_FeP_2_O_7_ three-phase system, introduced in Section 3.1 as a fundamental phase-purity challenge, is now being reevaluated through the lens of heterostructure design [65]. Armed with a quantitative understanding of each phase’s electrochemical contribution, researchers can pursue a new paradigm of “controlled impurity engineering.” In this paradigm, small amounts of the NFP phase are tolerated (or even deliberately introduced) to boost capacity, while the maricite content is maintained below the threshold that triggers significant performance degradation. Zhu et al. designed a heterogeneous NASICON-type composite cathode by integrating NFPP with Na_3_V_2_Ti(PO_4_)_3_ (NVTP), demonstrating reversible multielectron reaction chemistry [133]. The NFPP/NVTP heterointerface facilitates spontaneous charge redistribution and generates a built-in electric field that accelerates Na^+^ transport across the phase boundary, yielding a synergistic enhancement that surpasses the performance of either single phase (Figure 13b–d). The heterostructure design and interfacial synergistic effects are illustrated in Figure 13.

### 3.6. Comprehensive Comparison of Modification Strategies

Three main trends are seen in Table 2. (i) Combination approaches (high-entropy plus Fe vacancies, V/Al co-substitution, F/Si co-doping) consistently outperform single modification strategies, underscoring the synergistic nature of NFPP performance enhancement. (ii) The trajectory from Phase Adjustment to heterostructure design reflects a fundamental paradigm shift from passive acceptance of thermodynamic phase-equilibrium constraints to active design of favorable multiphase architectures. (iii) Mechanistic understanding of modification strategies progressed from empirical description to quantitative DFT-based elucidation. Computed values of band gaps, formation energies, and Na^+^ diffusion barriers have become standard reference metrics for guiding optimization. It should be noted that the capacity and cycling values reported in Table 2 were obtained under different current densities, voltage windows, carbon contents and electrode loadings and should therefore be compared qualitatively as trends rather than as directly comparable absolute numbers.

## 4. Summary and Outlook

### 4.1. Present Status

After over a decade of intensive research, NFPP has emerged as one of the most commercially promising cathode materials for sodium-ion batteries. This section summarizes the core conclusions of the present state of research.

(1) The structural foundation. NFPP possesses an outstanding Na^+^-storage architecture: a three-dimensional open framework of interconnected diffusion channels, an ultralow volume change below 4%, and a stabilization effect induced by the mixed polyanion matrix. Together, these features provide the physicochemical basis for its exceptional cycling stability and rate capability.
(2) Phase purity as the central challenge. The three-phase thermodynamic competition among NFPP, maricite-NaFePO_4_, and Na_2_FeP_2_O_7_ during synthesis is the fundamental bottleneck limiting NFPP electrochemical performance. Non-stoichiometric regulation combined with precursor innovation has proven the most productive path to date. Dual-iron-source engineering and self-sacrificial strategies have yielded phase purities as high as 96 wt.%.
(3) From trial-and-error to mechanism-driven design. NFPP doping is progressed from empirical screening to rationally designed, mechanism-guided strategies. Notable breakthroughs include Mo orbital delocalization engineering, V^3+^ coordination engineering (150.7 mAh g^−1^, 487 Wh kg^−1^), and the high-entropy cocktail effect. Together, these advances have crossed the theoretical capacity ceiling once thought insurmountable for the NFPP family.
(4) Anion and defect engineering as a new frontier. Oxygen vacancy tuning and anion substitution (SiO4^4−^, F^−^, and F/Si co-doping) define the 2025–2026 research frontier. These strategies extend the modification paradigm from the cation center to the anion framework. F/Si co-doping, which achieves a mere 1.5% volume change, signals the tantalizing possibility of genuinely zero-strain NFPP cathodes.
(5) A paradigm shift in phase-architecture thinking. Heterostructure and dual-phase design strategies mark a conceptual pivot from pursuing phase purity to harnessing multiphase synergy. This shift opens an alternative design space beyond the reach of single-phase optimization.

### 4.2. Future Research Directions

Direction 1: Rational phase purification—from empirical to predictive. Predictive control of phase purity demands complete thermodynamic and kinetic phase diagrams for the NFPP–NFP–NFPO ternary system. AI-driven high-throughput computational screening, when coupled with in situ characterization (environmental TEM and synchrotron XRD/XAS), can track phase evolution during synthesis in real time. This approach would identify optimal synthesis parameters without exhaustive experimentation. Novel precursor chemistries provide additional routes for kinetic phase-purity control: multi-iron-source synergy, pH-gradient regulation, and template-assisted crystallization.

Direction 2: Multi-scale structural design. Future NFPP development must span structural optimization at each relevant length scale. At the atomic scale, doping and defect design should follow molecular orbital theory and ligand field theory. At the nanoscale, quantitative control is needed over carbon-coating thickness, graphitization degree, and covalent bonding at the carbon–NFPP interface. The microscale demands optimization of secondary particle size distribution, tap density, and compaction density to maximize volumetric energy density. At the electrode scale, gradient pore-structure engineering for thick electrodes (areal loading > 10 mg cm^−2^) bridges material-level and device-level performance.

Direction 3: Performance and safety under extreme conditions. Critical gaps persist in understanding and optimizing NFPP performance under demanding conditions. At low temperatures (<−20 °C), Na^+^ desolvation becomes the rate-limiting step. At high temperatures (>60 °C), Fe dissolution and structural degradation mechanisms must be elucidated and mitigated through doping stabilization strategies. Under fast-charging conditions (10C/20C), thermal management emerges as the central concern. Standardizing safety-testing protocols (nail penetration, overcharge, external short-circuit, and hot-box tests) is imperative. So too is the accumulation of calendar-life and float-life data, which are almost entirely absent in the literature.

Direction 4: Sustainability and the full life cycle. Developing aqueous and green synthesis routes for NFPP is crucial for maximizing the environmental benefits of sodium-ion batteries. Minimizing organic solvent use and reducing calcination energy are key targets. NFPP-specific recycling technologies must also be developed. Because NFPP contains no expensive metals, such as Co or Ni, its recycling economics differ fundamentally from those of LIB cathodes. Direct regeneration methods (Na/Fe replenishment followed by re-calcination) may prove more economically viable than hydrometallurgical element recovery. A comprehensive cradle-to-grave life-cycle assessment (LCA), benchmarked against LFP-based LIBs, would provide a definitive environmental case for NFPP commercialization.

Direction 5: Cross-system applications and novel architectures. The NFPP framework may extend beyond sodium. Its three-dimensional open channels could accommodate the larger K^+^ radius, making potassium-ion batteries (KIBs) a promising target. Preliminary exploration in this direction warrants attention. The compatibility of NFPP with oxide and sulfide solid electrolytes in all-solid-state battery configurations demands systematic investigation. NFPP also holds potential in aqueous sodium-ion batteries. Here, the polyanion framework resistance to hydrolysis enables ultra-safe, ultralow-cost energy storage. High-throughput DFT screening should guide the combinatorial substitution of transition metals and anions. This approach could systematically expand the NFPP-derived structural family and uncover novel compositions that outperform the parent phase.

## Figures and Tables

**Table 1:** Comparison of NFPP synthesis methods [95,96,97,98,99,100,101,102].

Synthesis Method	Brief Principle	Advantages	Disadvantages	Commercialization Prospects
Solid-Phase Method	Solid-phase ball milling → high-temperature calcination	Simple process, low equipment cost; directly scalable to ton-scale batch production.	Difficult to achieve atomic-scale homogeneous mixing of precursors; poor carbon coating uniformity (typically rated “poor”); broad product particle size distribution (500 nm–5 μm); electrochemically inert maricite-NaFePO_4_ impurity phase readily forms.	★★★ (Suitable for low-cost mass production in low-end energy storage applications)
Sol–Gel Method	Solution chelation → gelation → calcination	Enables atomic-scale homogeneous mixing; lower calcination temperature (500–600 °C); excellent carbon coating uniformity; fine and uniform product particle size (200–500 nm); outstanding electrochemical performance.	High solvent consumption; low per-batch yield (gram-scale); gel drying and aging takes several days; high waste-liquid treatment cost; difficult to scale up industrially.	★★ (Primarily used for fundamental laboratory research and doping mechanism exploration)
Spray-Drying Method	Atomization → drying → calcination	Controllable particle morphology (spherical secondary particles); high tap density (~1.2–1.5 g/cm^3^); excellent carbon coating uniformity; good scalability (fully validated at kilogram scale); best overall performance.	High equipment investment; process parameters (solid content/inlet–outlet temperature/atomization pressure) require precise control; radial migration of carbon precursor during spray drying may cause inhomogeneous distribution with a carbon-depleted core and carbon-enriched shell.	★★★★ (Most promising for industrialization; multiple Chinese enterprises have initiated pilot-scale and mass production)
Solution Combustion Method	Solution self-propagating combustion reaction	Extremely fast reaction (<1 h); low synthesis temperature (300–500 °C); capable of producing ultrafine nanoporous particles (50–200 nm) with high specific surface area.	Uncontrollable reaction process (combustion front temperature can exceed 1000 °C while overall furnace temperature remains low); poor batch-to-batch consistency in product crystallinity and phase purity; difficult to scale up.	★★★ (Targeting specialty nanomaterial applications; batch production consistency requires further breakthroughs)
Electrospinning Method	High-voltage electrospinning → calcination	Capable of producing one-dimensional nanofiber morphology (diameter 100–300 nm); extremely high specific surface area; facilitates efficient electron transport along the fiber axis.	Extremely low yield (mg/h per g scale); high equipment cost; limited to laboratory-scale preparation; unable to meet the active material quantity requirements for practical battery assembly.	★★ (Fundamental research targeting specialty application scenarios such as flexible energy storage devices)
Ball-Milling–Spray-Drying Coupling	Ball mill homogenization → spray granulation → calcination	Combines the homogenization advantages of high-energy ball milling with the morphology control capability of spray drying; relatively high phase purity (good to excellent); uniform secondary particle size (5–10 μm); suitable for industrial-scale batch production; kilogram-scale preparation has passed preliminary engineering validation.	Two-step process increases manufacturing cost and total processing time; ball milling may introduce grinding media impurities (e.g., ZrO_2_); high requirements for precursor batch stability.	★★★★★ (Preferred technical route for industrial-scale mass production; achieves optimal balance between overall performance and scalability)

The number of ★ indicates the degree of commercial potential.

**Table 2:** Summary of NFPP modification strategy–performance–mechanism correlations.

Modification Strategy	Representative Material/Formulation	Optimal Specific Capacity (mAh g^−1^)	Optimal Cycling Performance	Core Mechanism	Ref.
**Phase Adjustment Strategies**					
**Non-Stoichiometric Ratio Control**	Na_3.4_Fe_2.4_(PO_4_)_1.4_P_2_O_7_ (NaFePO_4_ content 1.2–1.6)	110.8 (0.1C) 50.5 (100C)	83.1% capacity retention after 14,000 cycles at 20C	2NaFePO_4_–Na_2_FeP_2_O_7_ solid-solution model; elimination of maricite impurity	[115]
**Dual-Iron-Source Engineering**	FePO_4_ + FeC_2_O_4_ (kilogram-scale-up)	115.5 (0.1C) 74.3 (50C)	13 Ah pouch cell stable over 1000 cycles	Dual-iron-source synergistically regulates solid-state reaction pathway; suppresses maricite nucleation	[68]
**Self-Sacrificial Precursor**	Oxalate coordination (complete volatilization)	110.8 (0.1C) 101.5 (10C)	99.75% capacity retention after 2000 cycles	Amorphous precursor-mediated crystallization; bypasses thermodynamically competing phases	[116]
**Rietveld Quantitative Phase Analysis**	Na:Fe = 4:2.9 (optimal ratio)	110 (0.1C)	—	First quantitative deconvolution of electrochemical contributions from three phases; 96 wt.% NFPP highest phase purity	[114]
**Defect Engineering**					
**Fe Vacancy + High-Entropy Synergy**	Na_4_Fe_2.65_(NiCrMgCoMn)_0.027_(PO_4_)_2_P_2_O_7_	122 (0.1C)	>15,000 cycles ultra-long cycling	Fe vacancies widen Na^+^ channels; high-entropy stabilizes crystal structure	[67]
**Oxygen Vacancy Regulation (2026 VIP)**	Na_4_Fe_2.79_(PO_4_)_2_P_2_O_7_(NFPP-2.79)	89.5 (10C)	2C/1500 cycles, capacity retention 96.42%	Oxygen vacancy introduces donor level; weakens Na–O bond; diffusion barrier 0.53 → 0.38 eV	[54]
**High-Valence Cation Doping**					
**V^3+^ Doping (Classic Work)**	Na_3.94_Fe_2.9_V_0.06_(PO_4_)_2_P_2_O_7_/C	123.4 (0.1C)	20C/10,000 cycles, capacity retention 81.65%	V^3+^/V^4+^ electrochemical activity; V–O bond stronger than Fe–O, enhancing structural rigidity	[90]
**V^3+^ Coordination Engineering (Nat. Energy)**	Na_3.4_Fe_2.4_V_0.6_(PO_4_)_2_P_2_O_7_(NFV_0.6_PP)	150.7 (0.1C) 92.0 (20C)	5C/10,000 cycles, capacity retention 82.1%; 487 Wh kg^−1^	V^3+^ precisely occupies Fe2 site; activates inert Na sites; 3.4 Na^+^ reversible; single-phase reaction, no O_2_ release	[66]
**Mo^6+^ Doping (Orbital Delocalization)**	Na_4_Fe_2.91_Mo_0.09_(PO_4_)_2_P_2_O_7_	130.74 (0.1C)	50C/10,000 cycles, capacity retention 87.23%; 224.8 Wh kg^−1^	Mo 4d orbital delocalization; band gap 2.84 → 2.34 eV; Mo^6+^/Mo^4+^ reversible	[126]
**V^3+^/Al^3+^ Co-Substitution**	V/Al-NFPP (Fe-site co-substitution)	120 (0.1C) 72 (10C)	5C/1000 cycles, capacity retention 81%	Band gap 2.99 → 1.53 eV; energy barrier 0.529 → 0.398 eV; synergy surpasses single doping	[55]
**Low-Valence Transition Metal Doping**					
**Mn^2+^/Ni^2+^ Doping**	Mn-NFPP/Ni-NFPP	Mn: 92 (0.1C) Ni: ~85 (0.1C)	Mn: 100 cycles, capacity retention 99.5%	Suppresses maricite phase; improves Fe–O coordination; anionic redox	[56]
**High-Entropy Doping**					
**Five-Component High-Entropy (First Report)**	Na_4_Fe_2.85_(NiCoMnCuMg)_0.03_(PO4)2P2O7	122 (0.1C) 85 (50C)	82.3% capacity retention at 10C after 1500 cycles	Configurational entropy stabilization effect; cocktail effect; multi-element synergy	[130]
**Anion Substitution**					
**SiO_4_^4−^ Doping (Anion Substitution)**	Si-NFPP (preferential P1 site substitution)	119.4 (0.1C)	84.2% capacity retention at 50C after 5000 cycles	Preferential P1 site substitution; expanded a-axis lattice; enhanced inductive effect	[62]
**F^−^ Doping (Interstitial Site)**	F-NFPP (F intercalation at interstitial sites)	105.9 (1C)	86.2% capacity retention at 1C after 1000 cycles	Altered P_2_O_7_ dimer coupling distortion mode; reduced bandgap; elevated voltage plateau to 3.35 V	[132]
**F/Si Co-Doping (Dual-anion)**	NFPP-0.1F/0.05Si	119.6 (0.1C) 67.7 (10C)	80.86% capacity retention at 50C after 4000 cycles	Generation + stabilization of oxygen vacancies; volume change only 1.5%; synergy exceeds sum of single dopants	[63]
**Heterostructure and Dual-Phase Design**					
**Symbiotic Heterostructure**	Na_4.5_Fe_3.5_(PO_4_)_2.5_(P_2_O_7_)(NFP:NFPP = 0.5:1)	~130.5 (0.1C)	88% capacity retention at 20C after 11,000 cycles; ~400 Wh kg^−1^	NFP nanodomains → amorphization activation; NFPP matrix coating; heterointerface Na^+^ cross-transport	[64]
**NFPP/NVP Dual-Phase Composite**	NFPP-Na_3_V_2_(PO_4_)_3_(NVP)	125.2 (0.1C) 95.83 (10C)	75.3% capacity retention at 10C after 10,000 cycles	Heterointerface charge redistribution; NVP high ionic conductivity compensates NFPP; 1 + 1 > 2 synergistic effect	[65]

## Data Availability

The original contributions presented in this study are included in the article. Further inquiries can be directed to the corresponding authors.
